# Sensitive detection of extremely small iron oxide nanoparticles in living mice using MP2RAGE with advanced image co-registration

**DOI:** 10.1038/s41598-020-80181-9

**Published:** 2021-01-08

**Authors:** Joong H. Kim, Stephen Dodd, Frank Q. Ye, Andrew K. Knutsen, Duong Nguyen, Haitao Wu, Shiran Su, Simone Mastrogiacomo, Thomas J. Esparza, Rolf E. Swenson, David L. Brody

**Affiliations:** 1 Center for Neuroscience and Regenerative Medicine, Henry M. Jackson Foundation, Bethesda, MD USA; 2 Laboratory of Functional and Molecular Imaging, National Institute of Neurological Disorders and Stroke, National Institutes of Health, Bethesda, MD USA; 3 Neurophysiology Imaging Facility, National Institute of Mental Health, National Institute of Neurological Disorders and Stroke, and National Eye Institute, National Institutes of Health, Bethesda, MD USA; 4 Chemistry and Synthesis Center, National Heart, Lung, and Blood Institute, National Institutes of Health, Bethesda, MD USA; 5 Department of Biomedical Engineering, Washington University in St. Louis, St. Louis, MO USA; 6 Department of Neurology, Uniformed Services University of the Health Sciences, Bethesda, MD USA

**Keywords:** Biophysics, Neuroscience, Biomarkers, Medical research, Molecular medicine, Neurology

## Abstract

Magnetic resonance imaging (MRI) is a widely used non-invasive methodology for both preclinical and clinical studies. However, MRI lacks molecular specificity. Molecular contrast agents for MRI would be highly beneficial for detecting specific pathological lesions and quantitatively evaluating therapeutic efficacy in vivo. In this study, an optimized Magnetization Prepared—RApid Gradient Echo (MP-RAGE) with 2 inversion times called MP2RAGE combined with advanced image co-registration is presented as an effective non-invasive methodology to quantitatively detect T1 MR contrast agents. The optimized MP2RAGE produced high quality in vivo mouse brain T1 (or R1 = 1/T1) map with high spatial resolution, 160 × 160 × 160 µm^3^ voxel at 9.4 T. Test–retest signal to noise was > 20 for most voxels. Extremely small iron oxide nanoparticles (ESIONPs) having 3 nm core size and 11 nm hydrodynamic radius after polyethylene glycol (PEG) coating were intracranially injected into mouse brain and detected as a proof-of-concept. Two independent MP2RAGE MR scans were performed pre- and post-injection of ESIONPs followed by advanced image co-registration. The comparison of two T1 (or R1) maps after image co-registration provided precise and quantitative assessment of the effects of the injected ESIONPs at each voxel. The proposed MR protocol has potential for future use in the detection of T1 molecular contrast agents.

## Introduction

There is a major unmet need for molecular contrast MRI. At present, we have no way to accurately detect the vast majority of neuropathologies and pathophysiological processes in the living human brain. Brain biopsy is invasive and provides limited sampling. PET ligand development is a slow and challenging process, yielding excellent ligands for some pathologies and none for others. Furthermore, PET scans are expensive, have low spatial resolution, are of limited availability, involve specialized facilities, and require radiation isotope exposure. Biofluid markers do not provide anatomical specificity and suffer from the disturbance inherent in variable blood brain barrier permeability. In contrast, MRI is safe and widely available (in the developed world), providing high spatial resolution and anatomical specificity. However, MRI at present typically does not provide molecular specificity. A great deal of technical development will be required before molecular contrast MRI can be used to detect specific pathologies in the living human brain. These include MRI contrast agent optimization, blood brain barrier crossing, pathological target engagement, optimal MRI acquisition, brain clearance, safety, and repeatability of assessments, among others. Prior to deployment of these technologies in humans, optimization in experimental animals is required, including MR methods for detecting molecular contrast agents.

MR allows quantitative measurements of both T1 and T2 relaxation times in the brain. In most living tissues, the T1 times range from 200 to 2000 ms, whereas T2 is less than 100 ms^1^. MR contrast agents including iron oxide nanoparticles reduce both T1 and T2 in living tissue. Yet due to the intrinsic MR characteristics of living tissue, T2 shortening effects would have a maximum effect of about 100 ms whereas T1 shortening effects could be much larger. Thus, contrast agents utilizing T1-weighted MRI sequences have generally been preferred. In the detection of T1 contrast MRI, T1 weighted images are often used with good sensitivity to the effects of contrast agents^2–4^. However, T1 weighted images are affected by other factors such as RF coil sensitivity, field homogeneity, and the position of the subject relative to the MR signal receiver. These factors affect the reproducibility of MR data, which would prohibit detailed and precise analysis. Thus, T1 mapping is preferred for quantitative assessments of the effects of MR contrast agents. As an early step in this technical development program, we have focused on optimizing an MRI acquisition approach for quantitative T1 maps of in vivo mouse brain. We chose mice because of the extensive availability of mouse models of human neuropathological processes. For initial development, we have focused on the use of extremely small iron oxide nanoparticles (ESIONPs) having 3 nm iron core diameter as T1 MRI contrast agents^5^. Parallel work, to be reported elsewhere, involves alternative Gd-based^6–9^ and Mn-based^10–12^ T1 MRI contrast agents, as well as T2 MRI based contrast agents^13–16^.

There are many approaches to obtaining voxel by voxel T1 values (or T1 maps) of living tissues. T1 maps can be acquired from conventional inversion recovery MR pulse sequences, and the T1 maps from these conventional MR methods are widely used as reference standards. However, the inversion recovery methods are slow, even for the modified fast inversion recovery sequences^17^. Consequently, there have been many efforts to quantitatively assess the T1 value of living tissue using various alternative MR pulse sequences. The Look-Locker approach and modified versions are widely used for T1 mapping of heart, with fast scan times but relatively poor spatial resolution (more than one millimeter in-plane resolution with ~ 2 mm slice thickness) and target coverage (only one-three imaging slices)^18–20^. The variable flip angle approach utilizing two (or more) flip angles provide T1 maps with relatively short scan times, reasonable spatial resolution, and target coverage sufficient to cover the whole human brain^21–24^ but suffers from vulnerability to RF field inhomogeneity distortion. The magnetic resonance fingerprint (MRF) method provides T1 based on various MR signals along various MR parameters including repetition time, echo time, inversion delay time, and flip angle etc.^25–27^ but requires a comprehensive MR parameters dictionary, and relatively long scan times. MP2RAGE provides T1 mapping using two inversion delay time points after a single inversion RF pulse. Typically, a small flip angle, about 10 degree or less, is used^17,28^. For human brain MP2RAGE has (sub)millimeter isotropic voxel spatial resolution, whole brain coverage, and robustness to RF field inhomogeneity distortion with feasible scan times (about 10 min). In addition, the required parameters to obtain T1 values are well established for MP2RAGE in humans^28,29^.

**Figure 1:**
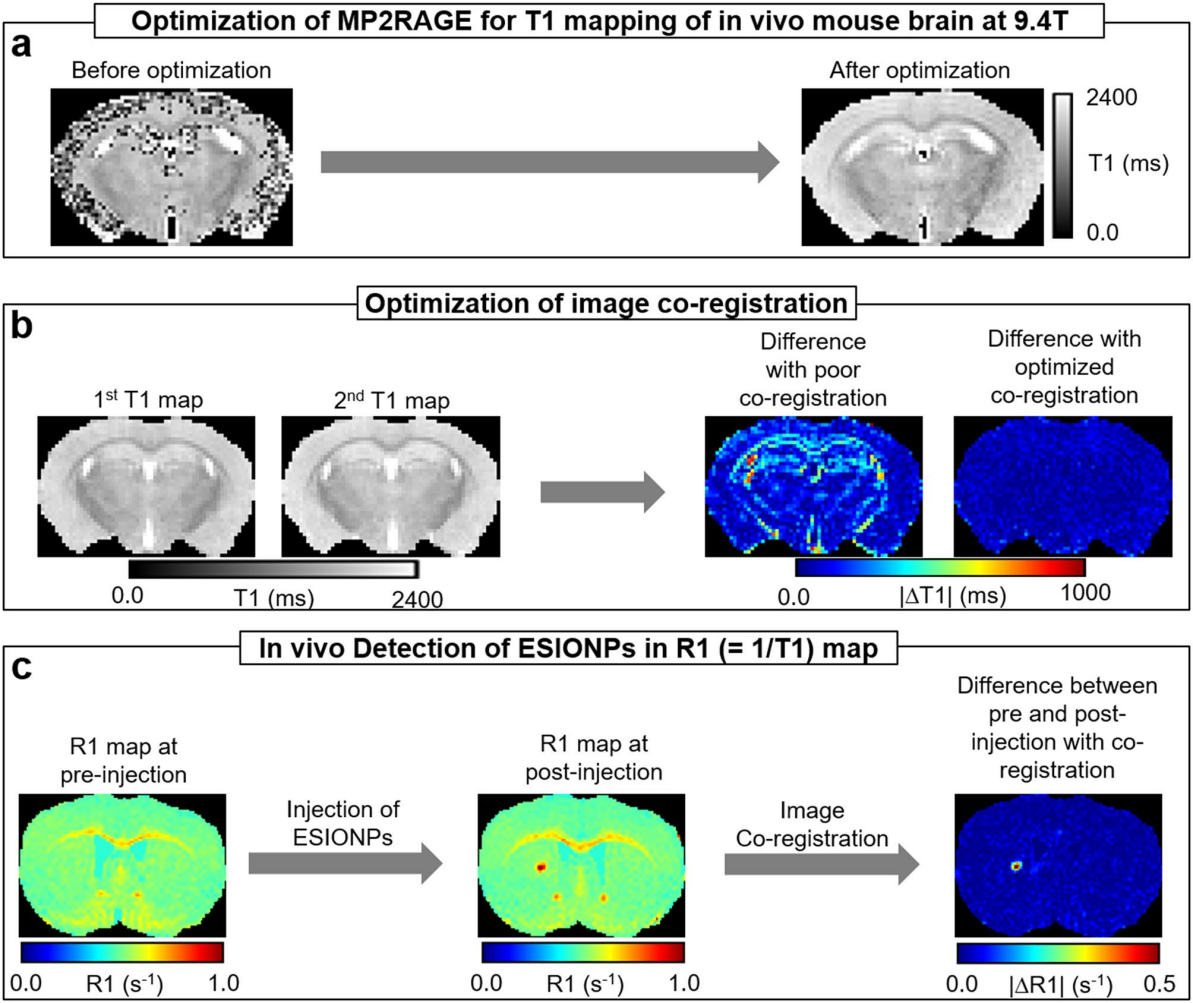
Schematic of experimental procedures. (**a**) Optimization of MP2RAGE for T1 mapping of in vivo mouse brain at 9.4 T (see Supplemental Figs. S1–S6 and Fig. 2). (**b**) Optimization of image co-registration (see Figs. 3, 4, 5). (**c**) In vivo detection of ESIONPs in R1 (= 1/T1) map (see Figs. 6, 7, 8).

Therefore, as briefly described in Fig. 1, we focused on optimizing T1 mapping MP2RAGE sequence, which was custom built, parameters at 9.4 T in mice for proof-of-concept molecular contrast studies. The T1 maps derived from MP2RAGE were precisely co-registered with the Advanced Normalization Tools (ANTs, http://stnava.github.io/ANTs/). The results were highly reliable T1 maps yielding sensitive and quantitative detection of injected ESIONPs in vivo.

## Results

### Optimization of T1 mapping MP2RAGE at 9.4 T

**Figure 2:**
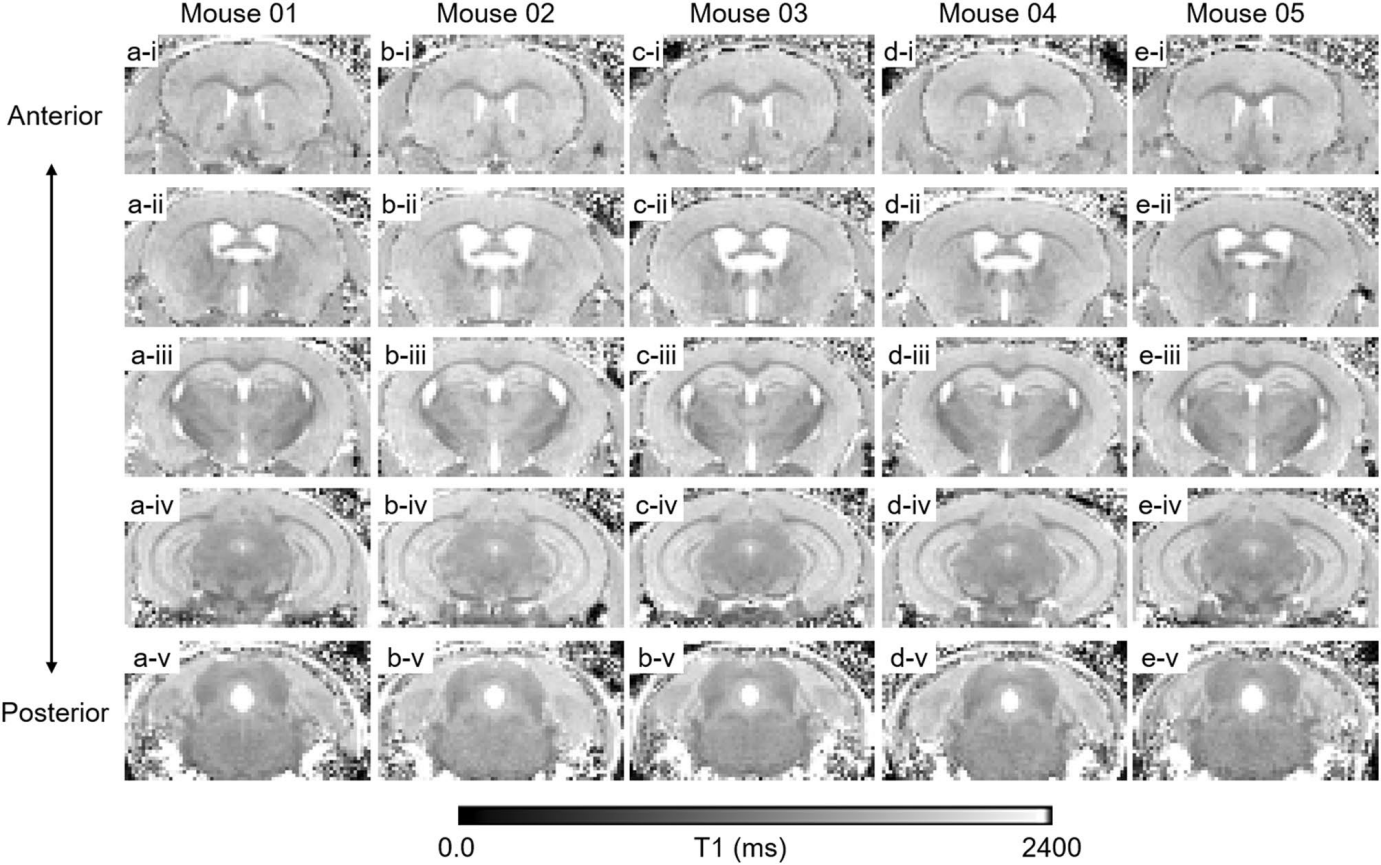
MP2RAGE-based T1 MRI maps from 5 control mice at 160 µm isotropic spatial resolution in vivo. (**a**–**e**) Each column represents an individual mouse. Row i. Anterior coronal slices at the level of the genu of the corpus callosum. Row ii. Anterior coronal slices at the level of the hippocampal commissure. Row iii. Coronal slices at the level of the anterior hippocampus. Row iv. Coronal slices at the level of the posterior hippocampus. Row v. Posterior coronal slices at the level of the 4th ventricle.

The major goal was to develop and test an efficient quantitative T1 MRI acquisition approach for detection of ESIONPs in the brains of living mice. We considered modified fast inversion recovery with optimized inversion delay time scheme^30^ and MP2RAGE^28^. Because detecting change in T1 signal before vs. after molecular contrast agent application was the goal, rather than absolute quantification of T1, we chose MP2RAGE as the most time-efficient option for in vivo imaging. While MP2RAGE sequences for human scanners have been widely used and extensively optimized, there has been relatively less work on MP2RAGE sequences for high field, small bore scanners used for high spatial resolution imaging of mice. Therefore, we performed a series of acquisition parameter optimizations in mice (Supplemental Figures S1–S6), and found that at 9.4 T, we could acquire 160 µm isotropic spatial resolution MP2RAGE whole brain coverage in vivo in 95 min (Fig. 2). MP2RAGE-based T1 maps were free of artifact, and relatively consistent from mouse to mouse, within the expected range of natural variability.

### Optimization of image co-registration

**Figure 3:**
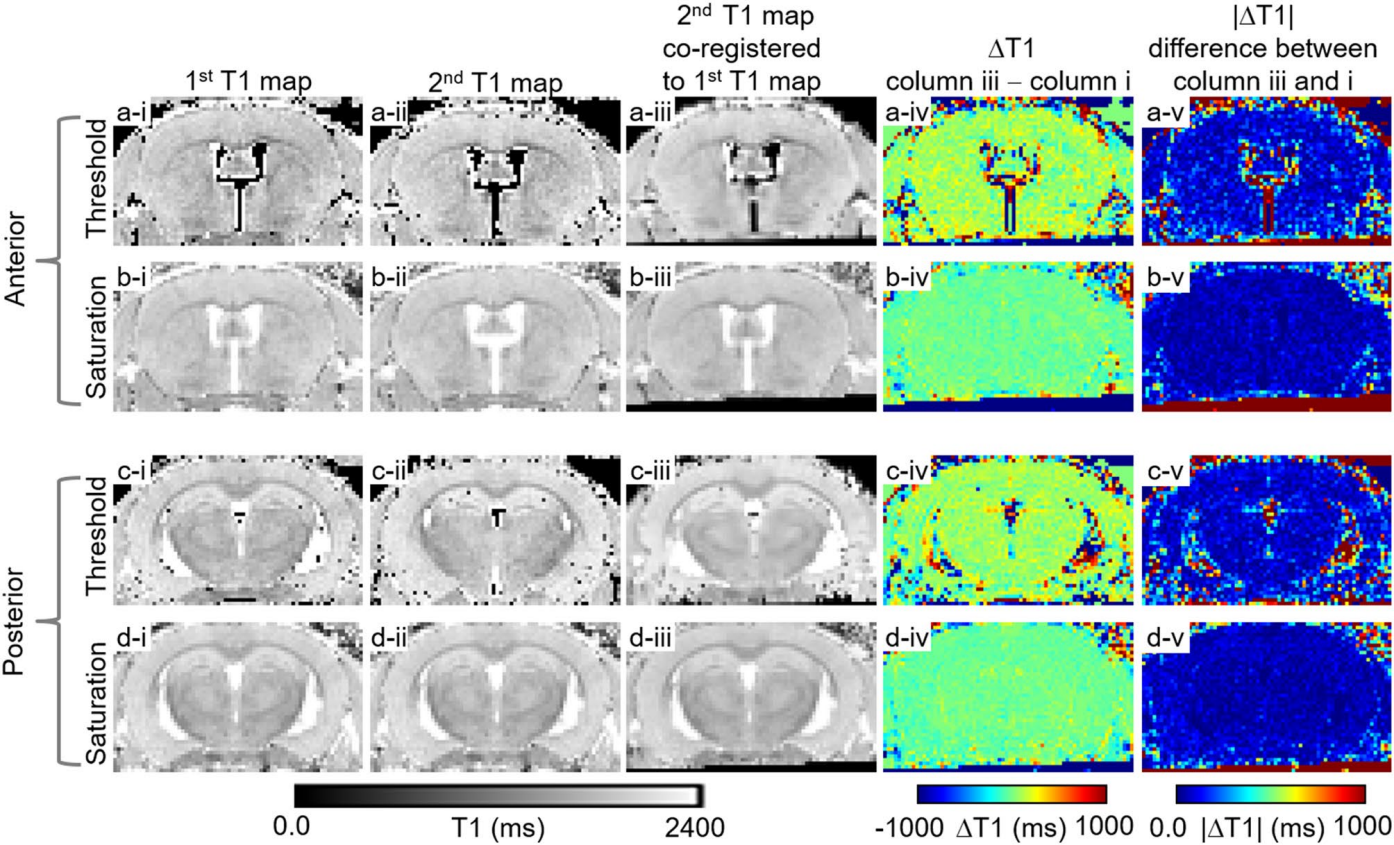
Comparison of threshold-based vs. saturation-based co-registration. column i. 1st T1 map from the first scan. column ii. 2nd T1 map from the second scan. column iii. 2nd T1 map co-registered to the 1st T1 map. column iv. subtraction result of 1st T1 map from 2nd T1 map co-registered to the 1st T1 map. column v. difference between 1st T1 map and 2nd T1 map co-registered to the 1st T1 map. row (**a**) and (**c**) MR results from threshold approach with extreme T1 values set to zero. row (**b**) and (**d**) MR results from saturation approach with extreme T1 values set to 300 and 2400 ms (300 ms as shortest limit and 2400 ms as longest limit). The saturation based co-registration approach produced better image co-registration.

**Figure 4:**
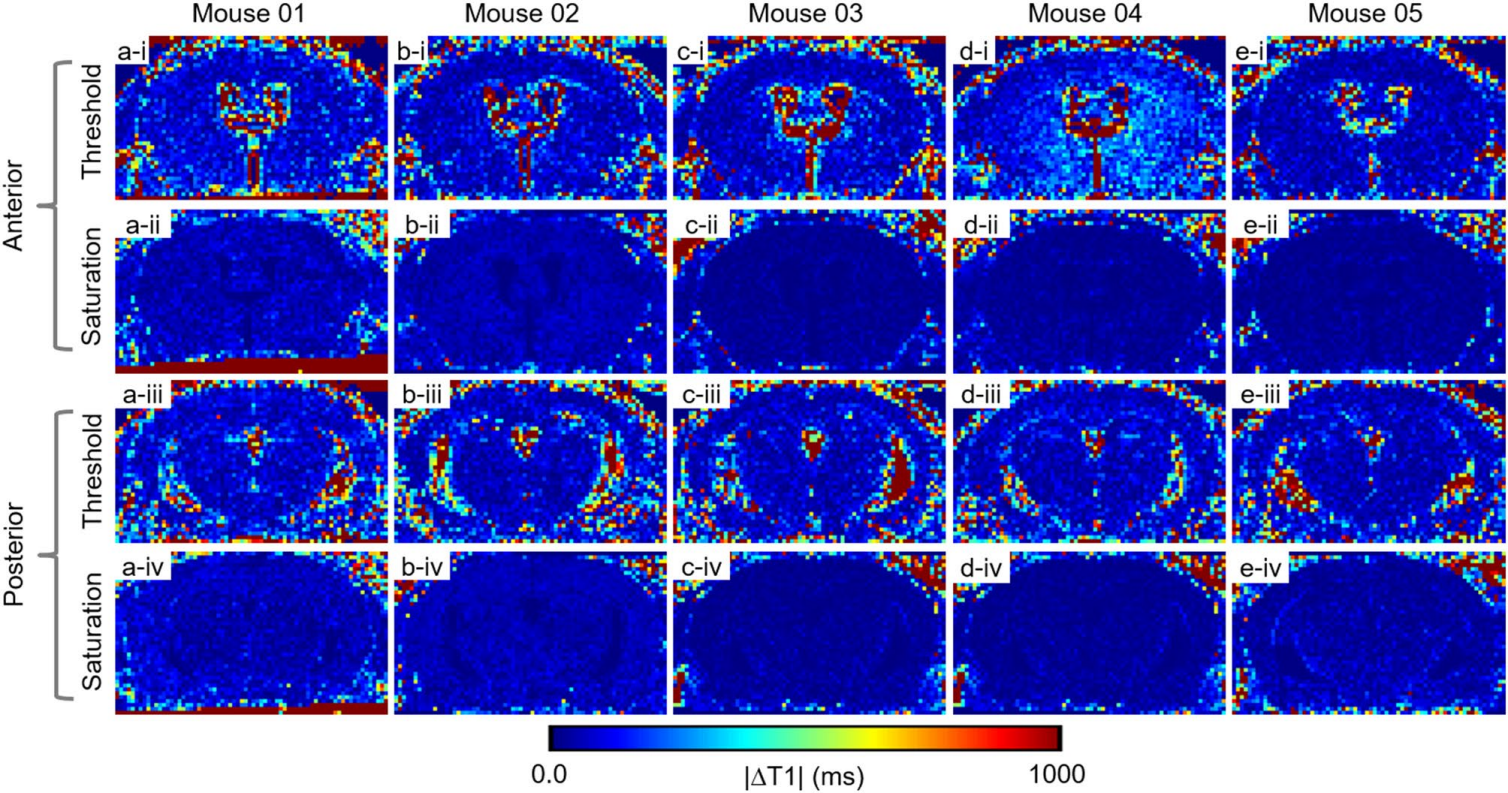
Absolute ΔT1 between first scan and second scan in 5 control mice. (**a**–**e**) Each column represents an individual mouse. Rows i, iii: absolute ΔT1 after co-registration using the threshold method. Rows ii, iv: absolute ΔT1 after co-registration using the saturation method. In all 5 mice, the saturation-based approach produced more accurate co-registration result than the threshold-based method.

The Advanced Normalization Tools (ANTs, http://stnava.github.io/ANTs/) is widely considered to be the state-of-the-art co-registration approach for MRI datasets^31^. We tested two approaches to co-registration using ANTs in mice: one based on setting voxels with extreme values of T1 to zero (threshold method), and one based on setting voxels with extreme values of T1 to the minimum or maximum T1 value found in the tissue of interest (saturation). We found that the saturation approach yielded dramatically better image co-registration results than the threshold approach (Fig. 3). Specifically, with the threshold approach there were substantial artifacts in and near the ventricles, with scattered areas of suboptimal subtraction throughout the brain (Fig. 3a-iv and –v, c-iv and-v) Instead, with the saturation approach, the difference between 2 separate MP2RAGE-based T1 maps was very close to zero, except for a few areas of artifact at the edges of the brain (Fig. 3b-iv and –v, d-iv and-v). The differences in T1 between scans were uniformly very low in gray matter and white matter, despite the lower T1 in white matter. The co-registration comparison was performed on 5 control mice. In all 5 mice, the co-registration was more accurate using the saturation-based approach than using the threshold-based method resulting in absolute differences close to zero (Fig. 4). The same conclusion was also observed from subtraction results (Supplemental Fig. S8).

### Reproducibility of T1 mapping using MP2RAGE

**Figure 5:**
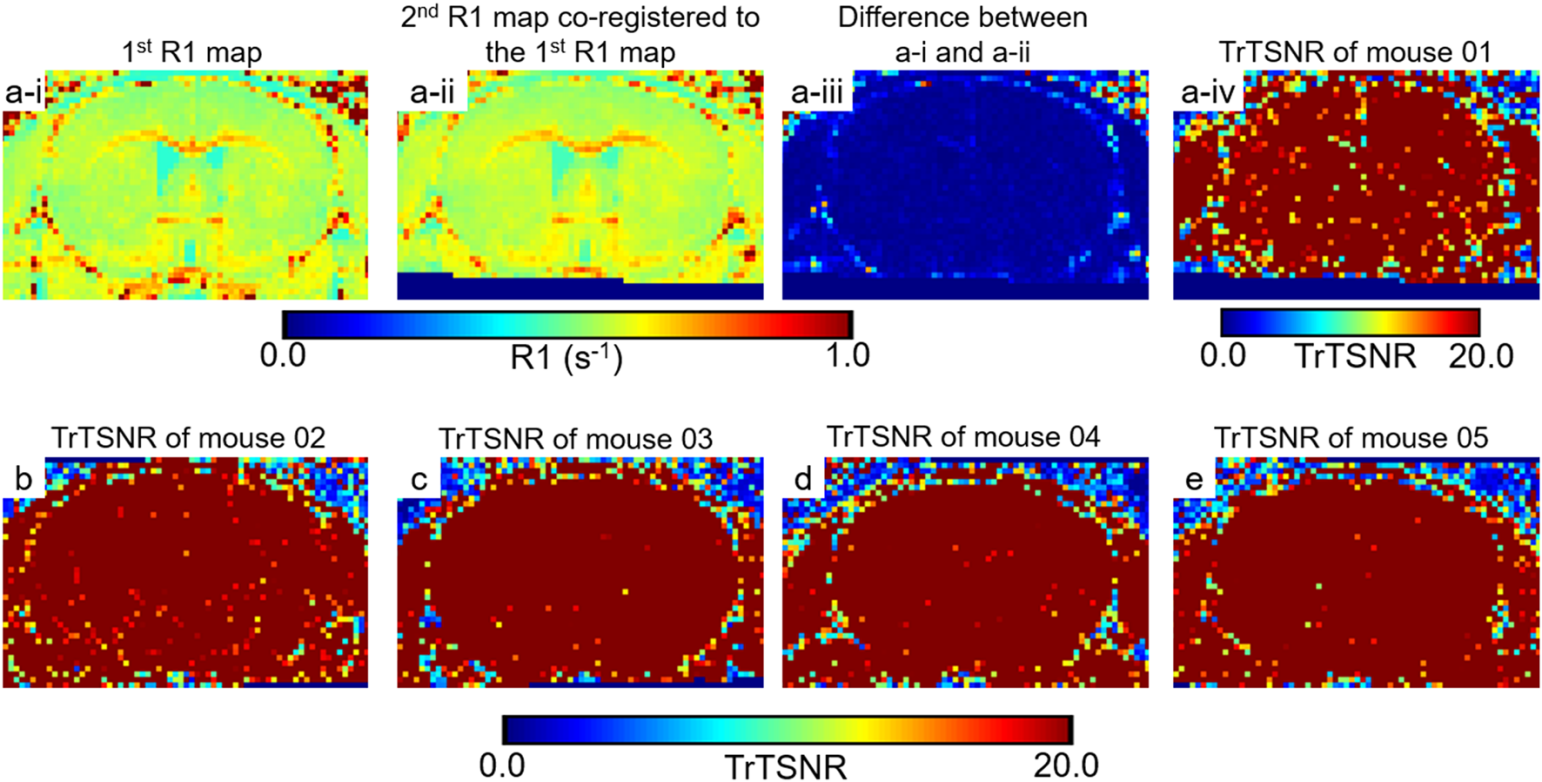
Reproducibility of R1 (1/T1) mapping. After saturation-based co-registration, all T1 (s) maps were converted into R1 (s^−1^) maps where R1 is defined as 1/T1. (**a-i**) R1 map using MP2RAGE. (**a-ii**) Repeat R1 map from a second acquisition in the same mouse, co-registered to the map from the first scan. (**a-iii**) ΔR1 map for each voxel, defined as R1 from scan 2–R1 from scan 1 after co-registration. (**a-iv**) test–retest signal to noise ratio (TrTSNR) map, defined at each voxel as the average R1 between the 2 scans divided by ΔR1. (**b**–**e**) TrTSNR maps for 4 additional mice. TrTSNR = 20 represents 95% reproducibility or 5% error. In all five control mice, TrTSNR of 20 or higher was observed.

We further quantified the accuracy and reliability of the MP2RAGE acquisition using test–retest signal to noise ratio (TrTSNR). We found that the MP2RAGE approach with saturation-based co-registration yielded TrTSNR of > 20 for nearly every voxel in 5 individual mice (Fig. 5). A TrTSNR > 20 indicates a > 95% reliability. TrTSNR was similar in gray matter and white matter, despite substantial differences in R1 (s^−1^, 1/T1). Areas of reduced TrTSNR were typically seen at the borders between the brain and skull, where partial volume effects may be prominent. Mouse 1 had slightly lower TrTSNR than the other 4 mice, but still had > 91% of voxels with TrTSNR above 20. These results indicated that the MP2RAGE approach is feasible for obtaining highly reproducible, quantitative T1 MRI scans in mice at high spatial resolution.

### Assessment of relaxivity of 3 nm ESIONPs

ESIONPs were produced and characterized at the NIH Image Probes Development Core facility using methods based on those of Kim et al.^5^. The iron oxide core size was 2.96 ± 0.84 nm (Supplemental Fig. S7b,c). Ligand exchange with phosphine oxide conjugated PEG 2000 was performed to make the ESIONPs soluble in aqueous solutions (Supplemental Fig. S7a). After ligand exchange, the hydrodynamic radius of the particles based on dynamic light scattering was 11 nm with − 1.32 mV zeta potential (Supplemental Fig. S7d,e). The relaxivity of the ESIONPs at 9.4 T was 2.9 s^−1^/mM iron.

### Detection of ESIONPs in vivo

**Figure 6:**
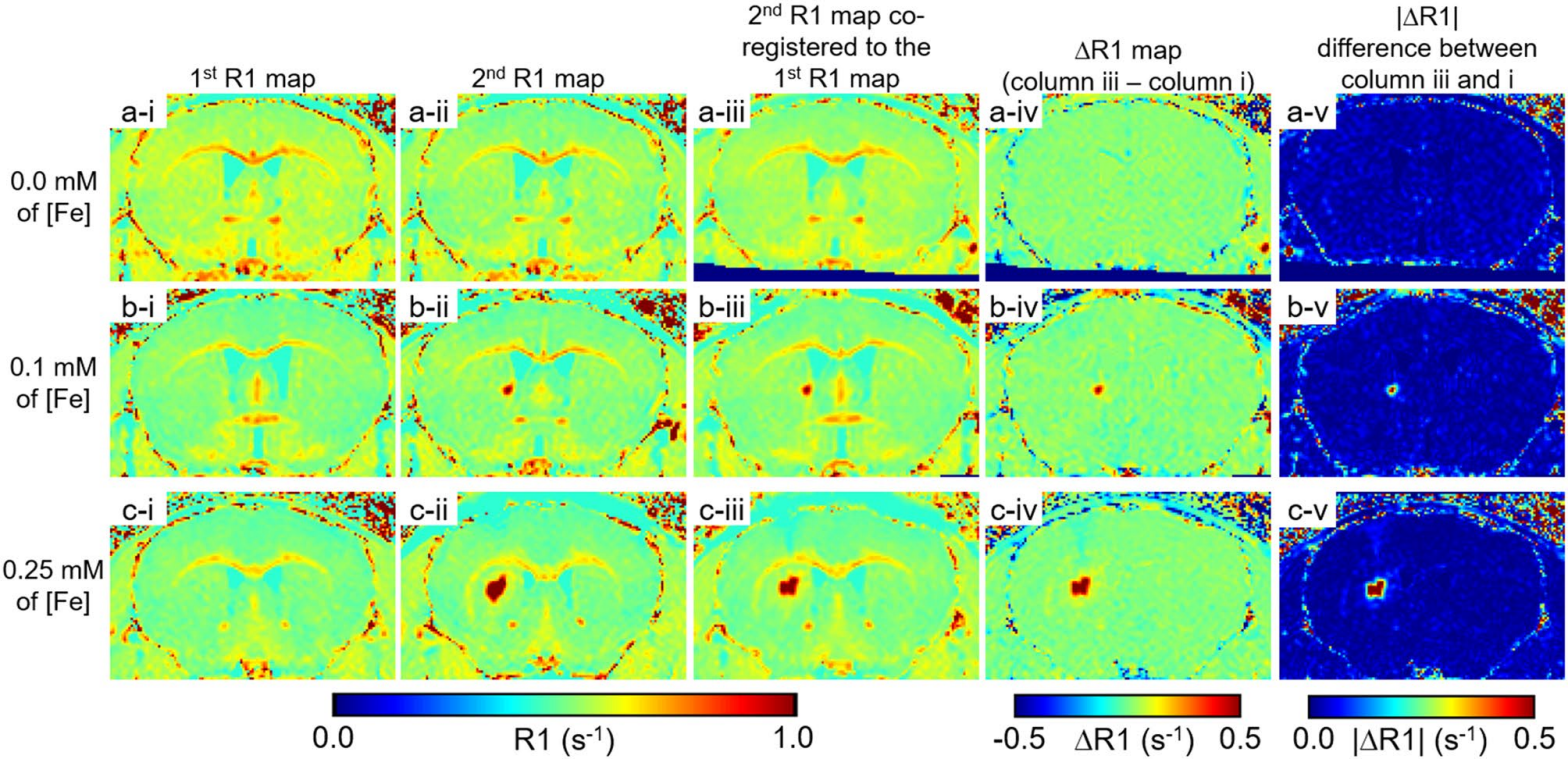
Detection of Extremely Small Iron Oxide Nanoparticles (ESIONPs) stereotaxically Injected into the Mouse Brain in vivo by MP2RAGE MRI. All maps were zero-filled from 160 × 160 × 160 µm^3^ to 80 × 80 × 80 µm^3^. (**a-i,b-i,c-i**) R1 maps from pre-injection MP2RAGE scans of 3 individual mice. (**a-ii**) R1 map from MP2RAGE scan after injection of saline. (**b-ii**) R1 map from MP2RAGE scan after injection of 1 µl of ESIONPs at 0.1 mM iron concentration (0.1 nMol iron). (**c-ii**) R1 map from MP2RAGE scan after injection of 1 µl of ESIONPs at 0.25 mM iron concentration (0.25 nMol iron). (**a-iii,b-iii,c-iii**) R1 maps after injection co-registered to pre-injection. (**a-iv,b-iv,c-iv**) ΔR1 maps of pre-injection scan from post-injection scans co-registered to the pre-injection. (**a-v,b-v,c-v**) Absolute ΔR1 maps between pre-injection scan and post-injection scans co-registered to the pre-injection. The R1 enhancing effect of injected ESIONP is evident in both ΔR1 and absolute ΔR1 (|ΔR1|) maps.

**Figure 7:**
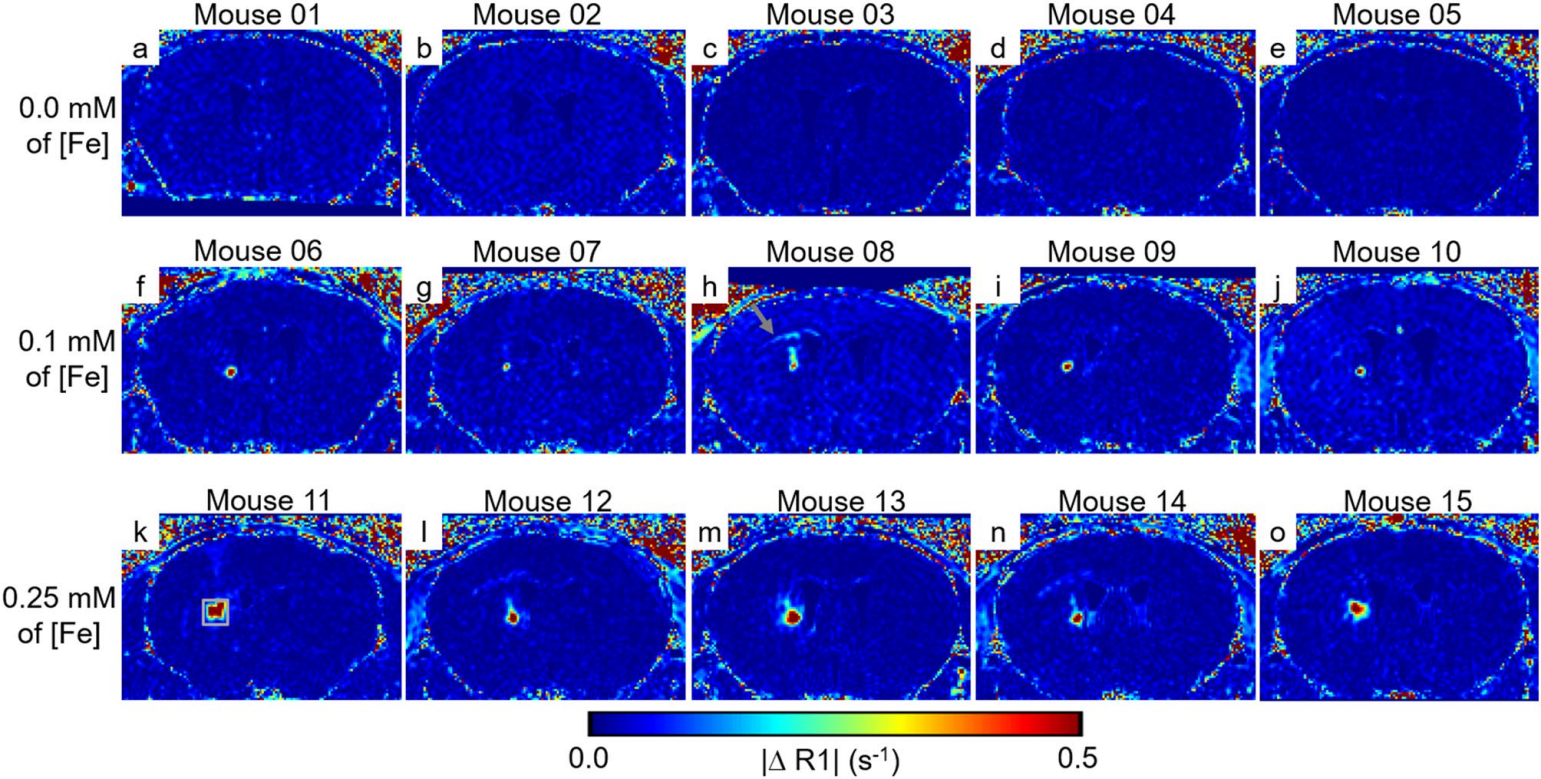
Consistent detection of ESIONPs after injection. All maps were zero-filled from 160 × 160 × 160 µm^3^ to 80 × 80 × 80 µm^3^. Each panel represents for individual mouse, total 15 mice. (**a**–**e**) **|**ΔR1| maps after injection of 1 µl of saline in 5 individual mice. (**f**–**j**) **|**ΔR1| maps after injection of 1 µl of ESIONPs at 0.1 mM iron concentration in 5 additional mice. (**k**–**o**) **|**ΔR1| maps after injection of 1 µl of ESIONPs at 0.25 mM iron concentration in 5 additional mice. The box in panel (**k**) indicates the region of interest used for quantitative analysis. Note that small amounts of signal change ipsilateral to injection indicated by arrow can be detected, likely due to variability in injection technique.

Next we assessed the detectability of ESIONPs in vivo using the MP2RAGE methods developed above. We took a ‘proof of concept’ approach and stereotactically injected the ESIONPs directly into the brain parenchyma. Two to three days before injection, each mouse was scanned using the MP2RAGE methods described above (Fig. 6a-i,b-i,c-i). Immediately after the injection of ESIONP, each mouse was scanned again (Fig. 6a-ii,b-ii,c-ii). The young adult wild-type mice used in these experiments tolerated the stereotactic injection and MRI scans back-to-back under anesthesia without detected adverse effects. The two scans were co-registered (Fig. 6a-iii,b-iii,c-iii) and subtracted to form ΔR1 maps (Fig. 6a-iv,b-iv,c-iv) and absolute ΔR1 (|ΔR1|) maps (Fig. 6a-v,b-v,c-v). The ESIONPs were clearly detectible on the MP2RAGE scans, and even more conspicuous on the both ΔR1 and |ΔR1| maps. Saline injection did not produce any detectible R1 artifact. The results were highly reproducible: in total 15 mice (5 mice per group), the injected ESIONPs were readily detectible in |ΔR1| maps (Fig. 7). In addition, a small change in R1 signal was detected along the injection tract (e.g. Fig. 7h,k,n) or spreading laterally in the ipsilateral corpus callosum (e.g. Fig. 7h,k,l,n). These findings likely arose from variability in the injection technique, and further emphasize the sensitivity of the subtraction method. The subtraction results also provided a similar level of ESIONPs detection (Supplementary Fig. S9).

We also examined the sensitivity of inversion recovery T1 weighted image (IRT1WI), a conventional MRI method, to the injected ESIONP. In general, the gray/white matter contrast was clear in IRT1WI, more evident at TI = 1300 ms than long TI (Supplementary Fig. S10). Yet IRT1WI either failed to detect low iron concentration ESIONP (Supplementary Fig. S12) or produced randomly scattered positive MR signal enhancements (Supplementary Fig. S13) even for control mouse (Supplementary Fig. S11), which had no relation to the injected ESIONPs. Thus, the MP2RAGE derived T1(or R1 = 1/T1) mapping approach with accurate image co-registration permitted reliable and pragmatically feasible detection of sub-nanomole quantities of ESIONPs in the brains of living mice.

### Estimation of detection limit of MP2RAGE for 3 nm ESIONPs

**Figure 8:**
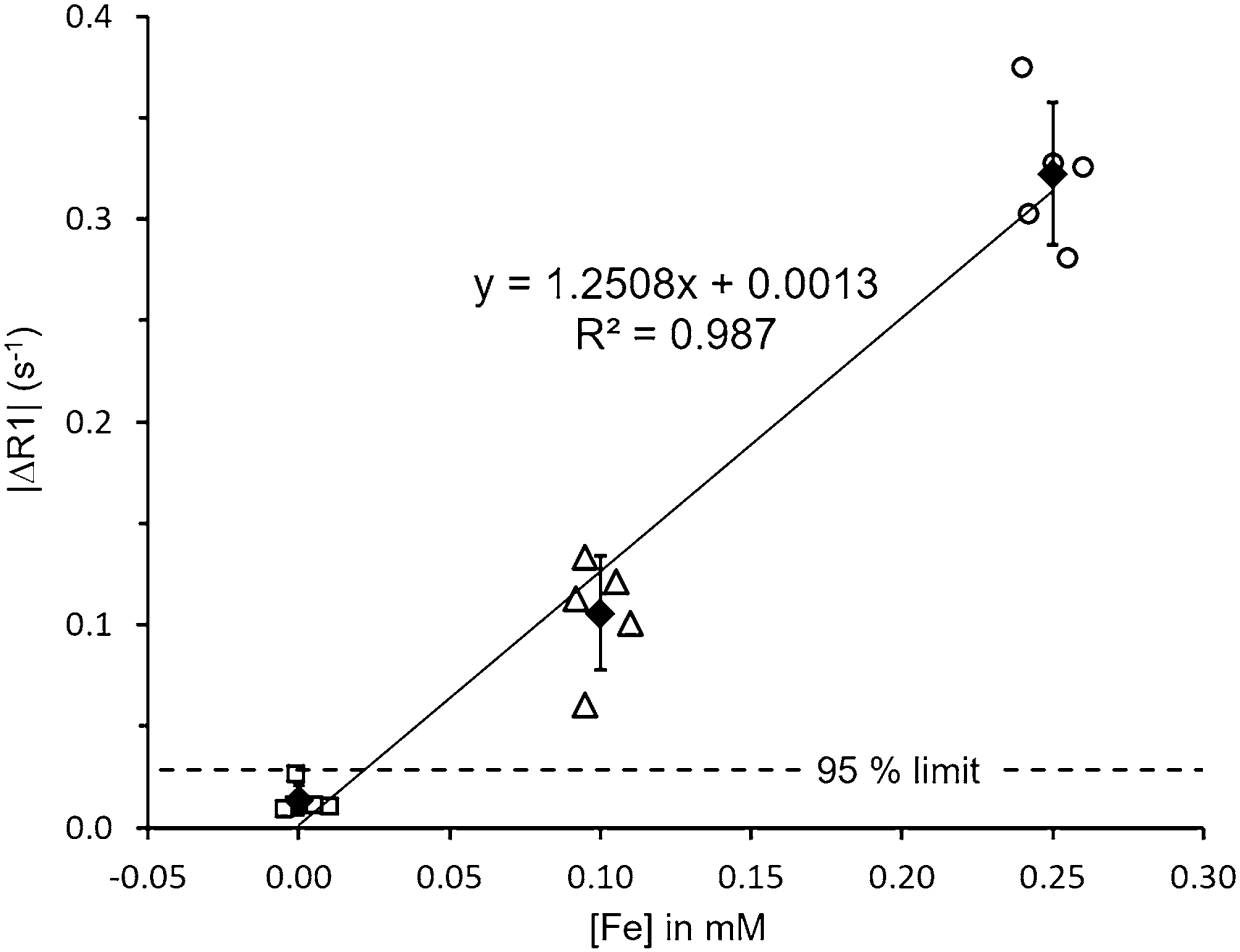
Quantitative Analysis of ESIONPs detectability. |ΔR1| in a 6 by 6 voxel region of interest around the injection site (shown in Fig. 7k) plotted as a function of injected iron concentration. Dashed line shows the 95% confidence interval around the |ΔR1| for saline injection (0.0 mM [Fe]), and indicates the theoretical detection limit. Solid line indicates the linear regression between |ΔR1| and [Fe]. Linear regression intersects the theoretical detection limit at about 0.03 mM [Fe].

To explore the probable lower limit of detectability of ESIONPs in the brains of living mice, we quantitatively analyzed the relationship between iron concentration and change in MP2RAGE-detected MRI signal (Fig. 8). First the R1 enhancement was quantified by region of interest analysis (ROI) and plotted as a function of iron concentration. The ROI size was chosen to cover the largest R1 enhanced brain region in this study, which is 6 by 6 voxel in 160 × 160 × 160 µm^3^ resolution. Then a linear regression between iron concentration and R1 change was calculated. The iron concentration at the intersection between the regression line and the 95% confidence band for the saline injected mice was defined as the detection limit. The linear regression between iron concentration and R1 change intersected the 95% confidence band for the saline injected mice at approximately 0.025 mM iron concentration, or 0.025 nmol of total iron. This indicates that the theoretical lower limit of detection is likely to be about 0.025 mM. With mouse-to-mouse variability similar to that at 0.1 mM iron, the lower limit of detectibly is likely to be about 0.05 mM.

## Discussion

In summary, we optimized MP2RAGE at 9.4 T to obtain quantitative T1 (or R1 = 1/T1) maps of the mouse brain in vivo with high spatial resolution and excellent reproducibility. The T1 mapping protocol combined with image co-registration was used to perform quantitative assessment of R1 enhancement induced by intracerebrally injected ESIONPs at a voxel-by-voxel basis. These results serve as demonstration of the feasibility of MRI methods that will be used for future brain molecular contrast agents. T1 mapping provides fully quantitative data that allows direct comparison between multiple sites, across studies, and over time in longitudinal analyses. An advantage of the MP2RAGE approach to T1 mapping is that it is readily translatable to human patients. MP2RAGE sequences are widely available and commonly used on human scanners, have excellent signal to noise, and can be performed quickly at high spatial resolution^29,32,33^.

The T1 shortening effects of 3 nm ESIONPs were readily seen with positive MR signal contrast, as in previous IONP-based MR studies^34,35^. The T1 shortening effects of IONPs have often been used to visualize pathologies in vivo by intravenous injection of IONPs in both pre-clinical^35–37^ and clinical studies^38,39^. Cell labeling with IONPs has enabled in vivo cell tracking in rodent T1 MR studies with intracranial injection of IONP-labelled cells^34,40^. Although the T1 relaxivity of small IONPs have been assessed using T1 (or R1) mapping methods in ex vivo phantoms, T1 weighted imaging has been mainly used to assess the T1 shortening effects of IONP in vivo^34–40^. However, T1 weighted image intensity can be affected by many factors other than the amount of IONPs. This would limit the quantitative reliability of T1 weighted imaging for IONPs in vivo. Considering the potential benefits of longitudinal studies, there have been efforts to utilize quantitative T1 mapping of the effects of IONPs in rodent studies^41,42^. The T1 (or R1) mapping provided objective quantification of IONPs induced R1 (= 1/T1) enhancement. The current study is fully based on T1 (or R1) mapping of the mouse brain in vivo. Furthermore, the current study has improved isotropic spatial resolution, 160 × 160 × 160 µm^3^, with whole brain coverage. The R1 map with whole brain coverage provides enhanced detectability of the MR contrast agent. In addition, the high spatial resolution enables advanced data analyses such as MR-to-histology correlation analysis^43^, which would provide strong validation of in vivo molecular contrast agent MR findings in future studies. Thus, the improvement of T1 mapping in the current study provides a strong foundation for future development of molecular contrast MRI.

In the ultimate instantiation of molecular contrast MRI, we envision that the patient would have an initial scan, be injected with the molecular contrast agent, and then be scanned again at an appropriate time later. The subtraction of the pre-contrast scan from the post-contrast scan would then yield a quantitative map of the retention of the contrast agent in the brain. Logically, in order to optimize the sensitivity to molecular contrast agents, the quantitative test–retest reliability of the MRI scan acquisitions should therefore be as high as possible. Stated another way, subtracting a first scan from a second scan should yield a map that is close to zero when there is no contrast agent in the brain. Because patients (and mice) may not be positioned in the scanner exactly the same way on each scan session, accurate co-registration of scans is required to make the subtraction map as close to zero as possible. Mimicking the in vivo human condition, in this study mice were taken out from MR scanner after pre-contrast MR scan, received ESIONPs through intracranial injection, and then repositioned for 2nd MR scan within one-week time frame. After image co-registration, both ΔR1 and |ΔR1| maps clearly visualized the ESIONP-induced R1 improvement allowing voxel-by-voxel analysis. Using an intravenous injection approach, it would be possible to collect MR data before and after injection of contrast without taking the subject out of the scanner. This would still not remove the requirement for image co-registration, since in vivo subject including human patients often move in the scanner. Furthermore, this would limit the application of molecular contrast agent MRI to specific studies like MR perfusion, MR angiography, or other acute studies. Longer-term studies with scans repeated several hours to days later would be required for most applications. While it is not known how long it will take to deliver molecular contrast agents across the blood–brain barrier, it is likely to take hours to days. Thus, the approach presented here involving image co-registration will likely be required for molecular contrast agent MR applications.

Our method to determine the detection limit of MP2RAGE for 3 nm ESIONPs was just one of several possible approaches. Quantification focusing more narrowly on the region with peak R1 change would increase the slope of the linear regression line, move the intersection point down to lower iron concentrations, yield a lower estimation of the detection limit. Furthermore, we have not yet assessed detectability for smaller regions at or below the size of single voxels. These will be important areas for future research.

Our study has several limitations. The current study mainly focused on brain parenchyma. Based on these results in mice, it may be more difficult to reliably detect pathologies that are close to the border between the brain and the skull, due to noise in the MP2RAGE measurements in these regions. Thus, meningeal pathologies or other superficial processes may be less suited to detection using this approach than parenchymal pathologies. Furthermore, we have not performed head to head quantitative comparisons between MP2RAGE-based T1 maps vs. T2 maps and T2* maps or quantitative susceptibility maps (QSM). It is likely that the optimal approach for human patients will represent a balance between sensitivity (likely to be higher for other methods) and specificity (likely to be best for T1 imaging methods). It is well known that most paramagnetic MR contrast agents including iron oxide nanoparticles have both T1 and T2 shortening effects^44,45^. However, T2 maps require long imaging times, and both T2* maps and QSM are vulnerable to field inhomogeneity, especially at air-tissue interfaces and in inferior areas of the brain^46^. If these issues can be overcome, it is likely that the same quantitative assessment with image co-registration used here with MP2RAGE data will be readily applicable to T2, T2* and QSM datasets.

One of the most critical steps in using MR contrast agent to detect specific brain pathology is the delivery of contrast agent to the brain. We have not yet determined whether the sub-nanomole concentrations of ESIONPs that are detectible using these MP2RAGE methods can be delivered to the brain non-invasively. In this study, we bypassed the blood brain barrier using direct injection into brain tissue. Extensive work around the world is focused on developing methods to get macromolecule-sized particles across the blood brain barrier (BBB) safely^47,48^. The ESIONPs used in this study had approximately 3.0 nm iron core size and 11.0 nm hydrodynamic size respectively. In addition to the iron core size, the hydrodynamic size of the ESIONPs is likely to affect both BBB penetration rate and T1 enhancing effects. The optimal ESIONP configuration for crossing the BBB and maximizing T1 enhancing effects remains to be determined. In addition, the ESIONPs will require further engineering for molecular specificity and low fouling.

In the future, reducing voxel size below 160 × 160 × 160 µm^3^ will increase detectability of low doses of highly localized MR contrast agents. High spatial resolution would also increase the precision of image co-registration, which will further improve the detectability of contrast agents by reducing noise introduced during co-registration. Smaller voxels size will require higher SNR through improved hardware, further optimized pulse sequences, and/or longer scans times with more averaging. The current study used 8.0 s TR, which is about four times longer than in vivo mouse brain T1 at 9.4 T. Considering that T1 represents the time when MR signal reaches 63% of equilibrium MR signal (or max. signal), TR can be reduced to 6 s, which will produce 95% of max. signal. The extra time can be used to perform additional averaging. In addition, there have been reports that cryoprobes increase SNR up to 40–50% without losing tissue characteristics in vivo ^49–51^. The human MR scanner hardware also has been improved including main fields of 7T^29,52^, multi-channel RF coils with up to 64 channels ^52^, and head only imaging gradient coil^53,54^. The employment of improved MR hardware will significantly increase SNR and spatial resolution.

In conclusion, we have optimized a quantitative T1 mapping MR protocol using MP2RAGE. Combined with image co-registration, the MR protocol can be used to quantitatively assess iron oxide nanoparticle induced R1 enhancement in the brain. These methods provide foundation building blocks for the future development of brain MRI molecular contrast agents.

## Methods

### Animals and ethics statement

All animal experiments in this study were approved by the National Institute of Neurological Disorders and Stroke (NINDS)/ National Institute on Deafness and Other Communication Disorders (NIDCD) Animal Care and Use Committee at the National Institutes of Health (NIH). All in vivo animal related procedures and methods were performed in accordance with the protocols approved by the Institutional Animal Care and Use Committee (IACUC) at the NIH.

C57BL6 female mice were purchased from Jackson labs at 10-weeks of age and used at 12-weeks of age. Three naïve mice were used for the MP2RAGE imaging parameter optimization study. The other 15 mice underwent two MP2RAGE MR scans with intracranial injection of ESIONPs or saline between scans.

### In vivo MRI on mouse brain

All MRI scans were performed on a 9.4 T scanner (Bruker, Ettlingen, Germany) with imaging gradient of 260 G/cm and Paravision 6.01 platform. A Bruker volume shape RF Transmit coil and 4- channel mouse brain surface shape signal receiver coil was employed. Mice were anesthetized with 90% oxygen/10% medical air gas mixture. Isoflurane level was maintained at 1.25–1.5% for in vivo MR scan and 5% for induction. Animals’ body temperature was maintained by blowing warm air with rectal temperature feedback. Mice underwent MR scan with custom written 3-dimensional MP2RAGE MR sequence having 8 s repetition time (TR), 3 ms echo time (TE), and six averages, requiring 95 min total scan time. The MP2RAGE MR data had 160 × 160 × 160 µm^3^ isotropic voxel size, zero-filled to 80 × 80 × 80 µm^3^, covering the entire brain. The MP2RAGE imaging parameters were optimized based on pilot experiments, (See supplement). Based on the optimization procedures, 9 degree flip angle, 640 ms segment duration with two segments (or two inversions for a k-space plane in 3-dimension), and 1.3/3.3 s inversion delay time points (TI) were employed to collect MP2RAGE derived MR data. Each mouse had two MP2RAGE MR scans at pre and post injection of ESIONPs or saline within one week. The post-injection MP2RAGE MR scan was started immediately after the injection.

### Intracranial Injection of ESIONPs

ESIONPs having 3 nm iron core and 11 nm hydrodynamic particle size were synthesized based on the methods described by Kim et al.^5^ with minor modifications (See the supplement for details). The ESIONPs were resuspended in 0.9% NaCl, 0.05% v/v Tween 80 producing 0.1 and 0. 25 mM of iron concentrations of ESIONPs including 0.0 mM of iron as control. Mice were anesthetized with 70% oxygen/ 30% medical air gas mixture containing 5% isoflurane in an induction box. After anesthesia was induced, mice were maintained at 1.5–2% isoflurane and put on a stereotactic platform in prone position. The head was secured to the stereotaxic frame using ear bars and a bite bar. An electrical heating pad was placed under the mouse to maintain temperature at 37 °C. A rectal temperature probe was used to monitor body temperature during anesthesia. Lubricant eye drops were applied on mouse eyes to prevent eyes from drying. Alcohol swabs and Povidone-iodine swabs were applied to the surgical site 3 times for antisepsis. A 4.0 mm length midline sagittal incision was made from bregma 2.0 to bregma − 2.0 to expose the skull. A hole was drilled at the coordinate x (right to midline) = 1.25 mm, y (posterior to bregma) = 1.0 mm using a hand drill (Braintree Scientific, Inc., Braintree, MA, USA) with a 0.45 mm diameter bit at 12,000 rotations per minutes (rpm). A 10 µl Hamilton syringe with 31 Gauge needle was mounted in a stereotaxic arm and lowered to 3.2 mm under the brain surface targeting the cerebral peduncle. The needle was left in place for 5 min. Then, 1.0 µl of the ESIONP solution or saline control was injected using a microinjector at a speed of 0.1 µl/min over 10 min. The needle was left in place for an additional 5 min post-injection then pulled out slowly. The incision was sutured using 4.0 silk suture. A subcutaneous (SQ) dose of Ketoprofen (100 mg/ml prepared in Lactated Ringers Solution, at a dose of 5 mg/kg body weight) was given once to mice for analgesia.

### Image and data processing

The inversion efficiency of the RF transmit coil employed for MP2RAGE was estimated by comparing MP2RAGE derived R1 (s^−1^, 1/T1) maps with R1 maps from inversion recovery (IR) (see supplemental Figs. S1–S3 for details). The estimated inversion efficiency, 0.85, was then used to calculate T1 maps for the remainder of the study. The MP2RAGE derived T1 maps were calculated with two different methods, threshold and saturation. In the threshold method, the voxels with extreme values of T1, which were background and ventricle regions, was set to zero, whereas in the saturation method, voxels with extreme values of T1 was set to either minimum (300 ms, background) or maximum (2400 ms, ventricle region) T1. After obtaining the T1 map at pre and post-injection of saline, the post-injection MP2RAGE MR data derived T1 maps (2nd T1 map) were co-registered to the pre-injection MP2RAGE MR data derived T1 map (1st T1 map) using advanced normalization tools (ANTs, http://stnava.github.io/ANTs/)^31^. The co-registration results based on threshold vs. saturation were compared. Based on the comparison, the saturation method was chosen to calculate the T1 maps from MP2RAGE MR data for the remainder of the study. The reproducibility of T1 maps were also quantitatively assessed using test–retest signal to noise ratio (TrTSNR) measure in the control group. TrTSNR was defined for each voxel as the average across the 2 measures divided by the difference between in the 2 measures. After co-registration of the 2nd R1 map to the 1st R1 map, the mean of two R1 maps was divided by the difference of two R1 maps for each voxel producing TrTSNR map. Region of interest (ROI) analyses were performed using 6 × 6 voxel in 160 × 160 × 160 µm^3^ voxel resolution hand-selected regions in a single slice. The general image handling including TrTSNR map, difference map, and subtraction map calculation was done using Image J version 1.52 s, https://imagej.nih.gov/ij/.

## Disclaimer

The views expressed here are those of the authors, and do not represent those of the NIH, Department of Defense, or other government agency.

## Supplementary Information

Supplementary Information.
