# Supplementary material for: Sensitive detection of extremely small iron oxide nanoparticles in living mice using MP2RAGE with advanced image co-registration
Source: Sci Rep. 2021 Jan 8;11:106. doi: 10.1038/s41598-020-80181-9 (PMC7794370; doi:10.1038/s41598-020-80181-9)
Supplement: Supplementary file 1 — Supplementary Information. [file 41598_2020_80181_MOESM1_ESM.docx]

**Supplements**

**Sensitive Detection of Extremely Small Iron Oxide Nanoparticles in Living Mice Using MP2RAGE with Advanced Image Co-registration**

Joong H. Kim^1,2^, Stephen Dodd^2^, Frank Q. Ye^3^, Andrew K. Knutsen^1^, Duong Nguyen^2^, Haitao Wu^4^, Shiran Su^2,5^, Simone Mastrogiacomo^2^, Thomas J. Esparza^1,2^, Rolf E. Swenson^4^, and David L. Brody^2,6*^.

^1^Center for Neuroscience and Regenerative Medicine, Henry M. Jackson Foundation, Bethesda, MD, USA.

^2^Laboratory of Functional and Molecular Imaging, National Institute of Neurological Disorders and Stroke, National Institutes of Health, Bethesda, MD, USA.

^3^Neurophysiology Imaging Facility, National Institute of Mental Health, National Institute of Neurological Disorders and Stroke, and National Eye Institute, National Institutes of Health, Bethesda, MD, USA.

^4^Chemistry and Synthesis Center, National Heart, Lung, and Blood Institute, National Institutes of Health, Bethesda, MD, USA.

^5^Department of Biomedical Engineering, Washington University in St. Louis, MO, USA.

^6^Department of Neurology, Uniformed Services University of the Health Sciences, Bethesda, MD, USA.

* corresponding author: David L. Brody Ph D, [david.brody@usuhs.edu](mailto:david.brody@usuhs.edu)

**Supplemental Materials and Methods**

Measurement of longitudinal relaxation rate (time) of in vivo mouse brain using modified fast inversion recovery MR at 9.4T;

The modified fast inversion recovery (MFIR)^1^ was employed with repetition time (TR, 8s), echo time (TE, 8ms), voxel = 150 x 150 x 500 µm^3^ and 3D gradient readout. The thick imaging slab (500 µm) was selected to ensure sufficient signal-to-noise ratio (SNR). The inversion recovery MR data were collected at 16 inversion delay time points (TI), which were 1.0, 1.1, 1.2, 1.3, 1.6, 1.9, 2.3, 2.6, 2.9, 3.3, 3.6, 3.9, 5.0, 6.0, 7.0, 7.5 seconds, (See **S-Figure 1**). Total scan time was 139 minutes per mouse. The longitudinal relaxation time constant (T1 (s)) was estimated using Bayesian analysis package (<https://bayesiananalysis.wustl.edu/>). The T1 values are also presented as longitudinal relaxation rate constant (R1 (s^-1^)) which is the inverse value of T1. Three naïve 12-week-old female C57BL6 mice were employed (s-mouse1, s-mouse 2, and s-mouse 3.) The estimated R1 maps of the three *in vivo* mouse brains were used as the reference R1 (or T1) values shown in **S-Figure 2**.

Optimization of Magnetization Prepared - 2RApid Gradient Echo (MP2RAGE) parameters at 9.4T;

3D-MP2RAGE^2^ was performed *in vivo* with TR (8 s), TE (3 ms), echo TR (eTR, 10 ms) and voxel size = 150 x 150 x 500 µm^3^. The same three mice (S-Mouse 1, S-Mouse 2, and S-Mouse 3) used for MFIR were also used for the MP2RAGE optimization. The MP2RAGE imaging parameters were optimized for i) estimated inversion efficiency, ii) flip angle, iii) segment duration, iv) first and second inversion delay time points (TI1 and TI2). Four imaging averages, requiring 17 min. scan time per mouse, were acquired for each part of the MP2RAGE parameter optimization study to ensure sufficient signal to noise ratio (SNR).

*i) Inversion efficiency*

The calculation of R1 values from MP2RAGE data requires the inversion efficiency as one of the parameters (as defined in equations A1.4 and A1.5 of Marques et al., Neuroimage 2010.^2^) However, inversion efficiency in MPR2RAGE cannot be directly measured. Instead, we calculated MP2RAGE-based R1 maps based on several plausible hypothesized values of inversion efficiency, and compared the MP2RAGE-based R1 maps with MFIR-based R1 maps obtained in the same mice, See **S-Figure 2**. We used the value of inversion efficiency that produced the closest match between the MP2RAGE-based and MFIR-based R1 maps. The MP2RAGE data for inversion efficiency were collected using the following parameters; 9 degree flip angle, 640 ms segment duration, and 1.3/3.3 sec for TI1/TI2. The three parameters were chosen based on optimization described below. R1 maps were calculated from the MP2RAGE data for hypothesized inversion efficiency 0.75, 0.8, 0.85, 0.9, and 0.95. The results are shown in **S-Figure 2 and 3**. Based on the results, the inversion efficiency of MP2RAGE was fixed to 0.85 for entire study. The match was not perfect, and the MP2RAGE-based R1 maps should be considered approximate, not precise, measures of tissue R1. We note that the inversion efficiency is highly dependent on the transmit RF coil. Thus the same transmit RF coil was employed for entire study.

*ii) Flip angle*

The MP2RAGE data for flip angle optimization were collected using following parameters; 640 ms segment duration, 1.3/3.3 second of TI1/TI2, and four flip angles (3, 5, 7, and 9 degrees). R1 maps were calculated using 0.85 inversion efficiency. The results are shown in **S-Figure 4**. The R1 of the three mouse brains were largely preserved from 3 to 9 degrees. The R1 maps from 3 degree flip angle had artifacts due to insufficient SNR. Based on this result, the flip angle was set to 9 degree for the entire study to ensure sufficient SNR for the target voxel size of 160 x 160 x 160 µm^3^ that would be used for the main study.

*iii) Segment Duration*

The optimization of segment duration for MP2RAGE data collection was done using following parameters; 9 degree flip angle, 1.3/3.3 second of TI1/TI2, and three segment duration times (160 ms, 320 ms, and 640 ms). The R1 maps, calculated using 0.85 inversion efficiency, are shown in **S-Figure 5**. In general, the R1 values are largely preserved from 160 ms to 640 ms duration time. A few scattered artifacts were seen in the short segment duration time scans. As shown in **S-Figure 5** panel a, higher signal would be expected with longer segment duration times. However, the segment duration time also affects the interval between the two inversion delay time points (TI1 and TI2). Thus, the TI1 and TI2 time points also need to be considered when optimizing the segment duration time.

*iv) Inversion delay time points (TI1 and TI2)*

The inversion delay time points were optimized using MP2RAGE data acquired with following parameters: 9 degree flip angle, 640 ms segment duration time, and TI1/TI2 (s) = 1.1/3.3, 1.3/3.3, 1.3/6.0, and 1.5/3.6. The results are shown in **S-Figure 6**. The TI1/TI2 (s) = 1.3/3.3 showed good quality R1 maps, whereas there were artifacts apparent with other TI1/TI2 acquisitions. The R1 maps with TI1/TI2 (s) = 1.1/3.3, column ii in S-Figure 6, suffer from overestimated R1 artifacts. The 1.1 sec TI1 is very close to null point of inversion recovery curve (see **S-Figure 1**) resulting in low SNR MP2RAGE images. The R1 maps with TI1/TI2 (s) = 1.5/3.6, column iii in **S-Figure 6**, suffer from underestimated R1 artifact indicated by arrows. Considering the 640 ms segment duration time, the 1.5 second TI1 is relatively close to TI2 (3.3 second) leaving insufficient inversion recovery between two inversion time points and underestimation of R1. R1 maps from TI1/TI2 (s) = 1.3/6.0, column iv in **S-Figure 6**, also suffer from similar artifacts seen from the R1 maps with TI1/TI2 (s) = 1.5/3.6. The TI2 (6.0 second) is very far from TI1 (1.3 second) deviating from the approximately linear inversion recovery region. Based on the results from **S-Figure 5 and 6**, the segment duration time and TI1/TI2 were fixed at 640 ms and 1.3/3.3 seconds.

Overall, the optimized MP2RAGE parameters for *in vivo* mouse brain imaging at 9.4T were 0.85 inversion efficiency, 9 degree flip angle, 640 ms segment duration with TI1/TI2 (s) = 1.3/3.3.

*Production and Characterization of Extremely Small Iron Oxide Nanoparticles*

Extremely small iron oxide nanoparticles (ESIONP) were produced using the methods detailed in Kim et al.^3^ and Na et al.^4^ with minor modifications, See **S-Figure 7**. Synthesis and ligand exchange ~~were~~ performed at the NIH Intramural Imaging Probe Development Center (IPDC), now known as the Chemistry and Synthesis Center (CSC). Reagents were obtained from the following sources: iron chloride hexahydrate (FeCl_3_·6H_2_O), 80% oleic acid, Poly(ethylene glycol) methyl ether (PEG750), tetrahydrofuran (THF) and dichloromethane were purchased from Sigma Aldrich. Sodium oleate was purchased from TCI. Hydroxyl-PEG-Azide 2k (OH-PEG-N_3_ or PEG2000-Azide) was obtained from Biochempeg.

ESIONP core sizes were measured using JEOL-200 CX electron microscope. Sample was placed on a formvar/carbon grid (FCF-200-Ni, Electron Microscopy Sciences) Images were taken of random fields at direct magnification of 73000x, 120kV. Core sizes were quantified using ImageJ software.^5^ The core size was 2.96 +/- 0.89 nm.

After ligand exchange with PEG2000-Azide, ESIONPs were dissolved in 0.9% NaCl, 0.05% v/v Tween 80 at a concentration of 1 mg/ml. Iron concentration was measured using a 96 well plate-based Ferene-S (3-(2-Pyridyl)-5,6-di(2-furyl)-1,2,4-triazine-5′,5′′-disulfonic acid) assay ^6^ as previously detailed, using a reference iron solution to produce a standard curve in triplicate. The results from the Ferene-S assay agreed well with results from Inductively Coupled Plasma-Optical Emission Spectrometry (ICP-OES).

Dynamic light scattering measurement of hydrodynamic particle size and zeta potential were performed using a Zetasizer (Nano ZS, Malvern). The size was approximately 11 nm and zeta potential was -1.31 mV. Size exclusion chromatography was performed using an AKTA Purifier with a Superose 6 column to assess the homogeneity of the particles. Gel filtration standards of globular protein size standards (Cat#1511902, BioRad) were run under identical conditions for reference. Under these conditions, the void volume is 9 ml, and aggregated particles would be expected to either not enter the column at all or run at the void volume.

*R1 Measurements of ESIONPs*

R1 measurements for the ESIONPs were performed using a series of dilutions. MFIR was used to measure R1 at 0.02, 0.04, 0.05, 0.06, 0.08, 0.1, 0.25, 0.5, and 1.0 mM iron concentrations of ESIONPs at 9.4T. The R1 (1/T1) of each ESIONP was estimated using Bayesian analysis package (<https://bayesiananalysis.wustl.edu/>). A linear regression of R1 (s ^-1^) vs. iron concentration was used to measure molar relaxivity (2.9 mM^-1^s^-1^).

**Supplemental Figures**

**S-Figure 1. Inversion recovery of in vivo mouse brain at 9.4T.** The inversion recovery MR data of *in vivo* mouse brain from three naïve adult mice are shown at 16 inversion delay time points, mean ± standard deviation (SD). The quantified intensity was normalized by the MR signal at 7.5 seconds inversion delay time. The red and blue boxes represent the two selected inversion delay time points for MP2RAGE. The width of each box represents segment duration time, 640 ms.

**S-Figure 2. Representative R1 maps of a naïve *in vivo* mouse brain. a-i, b-i:** R1 maps from modified fast inversion recovery at anterior (a-i) and posterior (b-i) coronal slices. **a-ii** – **a-vi:** R1 maps from MP2RAGE at an anterior coronal slice.  **b-ii** – **b-vi:** R1 maps from MP2RAGE at a posterior coronal slice. MP2RAGE data were acquired with 9 degree flip angle, 640 ms segment duration time, and TI1/TI2 (s) = 1.3/3.3. R1 maps were calculated using hypothesized inversion efficiency of 0.75 (column ii), 0.80 (column iii), 0.85 (column iv), 0.90 (column v), and 0.95 (column vi). The R1 of *in vivo* mouse brain derived from MP2RAGE increased with higher hypothesized inversion efficiency. The quantified R1 values are shown in **S-Figure 3**.

**S-Figure 3. Quantified longitudinal relaxation rate constant (R1 (s^-1^)) of three naïve *in vivo* mouse brains from modified fast inversion recovery (MFIR) and MP2RAGE.** The squares, triangles, and circles represent S-Mouse 1, S-Mouse 2, and S-Mouse 3 respectively. The error bars represent the standard deviations across voxels in each region of interest (ROI) histogram. **a.** Quantified R1 from genu of corpus callosum (GCC). **b.** Quantified R1 from mid-corpus callosum (CC). **c.** Quantified R1 from cortex. The R1 of *in vivo* mouse brains derived from MP2RAGE increased with higher hypothesized inversion efficiency in all brain regions. **d.** R1 differences between tissues: GCC vs. cortex (white diamonds) and GCC vs. CC (black diamonds). Compared to the R1 from MFIR, the MP2RAGE overestimated the R1 differences between different brain tissues. The overestimation of R1 differences was largely preserved for all hypothesized inversion efficiencies. Based on the results, an inversion efficiency of 0.85 for MP2RAGE was used for all R1 calculations in the entire study.

**S-Figure 4. Optimization of flip angle for MP2RAGE.** The R1 maps were calculated using 0.85 inversion efficiency. Acquisition parameters included 640 ms segment duration and TI1/TI2 (s) = 1.3/3.3. **row i:** R1 maps of three naïve *in vivo* mouse brains with flip angle 3 degrees. **row ii:** R1 maps of three naïve *in vivo* mouse brains with flip angle 5 degrees. **row iii:** R1 maps of three naïve *in vivo* mouse brains with flip angle 7 degrees. **row iv:** R1 maps of three naïve *in vivo* mouse brains with flip angle 9 degrees. Overall, the R1 values were well preserved from 3 to 9 degrees for all three mice. The R1 maps with 3 degree flip angle had random artifacts, indicated by arrows, due to low SNR. No evidence or R1 deviation was observed at 9 degree flip angle compared to R1 at lower flip angles.

**S-Figure 5. Optimization of segment duration for MP2RAGE.** The R1 maps were derived from MP2RAGE data using 0.85 inversion efficiency, 9 degree flip angle, and TI1/TI2 (s) = 1.3/3.3. **a.** 160 (blue box with solid line), 320 (gray box dotted line), and 640 ms (red box with dashed line) segment duration for inversion recovery acquisition of MP2RAGE data for *in vivo* mouse brain. **column i.** R1 maps from 160 ms segment duration. **column ii.** R1 maps from 320 ms segment duration. **column iii.** R1 maps from 640 ms segment duration. The R1 values were largely preserved for all three segment duration times, but noise was lower in with longer segment duration.

**S-Figure 6. Optimization of TI1 and TI2 for MP2RAGE.** The R1 maps were derived from MP2RAGE data using 0.85 inversion efficiency, 9 degree flip angle, and 640 ms segment duration time. **column i.** R1 maps from TI1/TI2 (s) = 1.3/3.3. The R1 maps with TI1/TI2 (s) = 1.3/3.3 show good quality of *in vivo* mouse longitudinal relaxation rate constant measures. **column ii.** R1 maps from TI1/TI2 (s) = 1.1/3.3. The R1 maps with TI1/TI2 (s) = 1.1/3.3 suffer from overestimated R1 artifacts, apparent as bright spots. **column iii.** R1 maps from TI1/TI2 (s) = 1.5/3.6. The R1 maps with TI1/TI2 (s) = 1.5/3.6 suffer from underestimated R1 artifact indicated by arrows. **column iv.** R1 maps from TI1/TI2 (s) = 1.3/6.0. Similar artifacts are seen from the R1 maps with TI1/TI2 (s) = 1.3/6.0.

**S-Figure 7. Characterization of Extremely Small Iron Oxide Nanoparticles (ESIONPs).** **a.** Photograph of the ESIONPs with polyethylene glycol coating and an azide functional group (ESIONP-PEG-Azide) suspended in 0.9% NaCl, 0.05% v/v Tween 80 solution. The ESIONPs were highly soluble in aqueous solutions. **b.** TEM Image of ESIONP cores. **c.** Close up view of the box in panel b indicating homogenous iron oxide core sizes. **d.** Dynamic light scattering (DLS) size distribution of ESIONP-PEG-Azide, mean value of 11.0 nm. **e.** Zeta potential of ESIONP-PEG-Azide, mean value of -1.31 mV. **f.** Size exclusion chromatography of ESIONP-PEG-Azide (red curve, red arrow) in human plasma with size standard references (gray curve, dotted arrows). The ESIONPs had homogenous hydrodynamic size and no significant fouling.

**S-Figure 8: ΔT1 of first scan from second scan in 5 control mice**. **a-e:** Each column represents an individual mouse. **Rows i, iii**: absolute ΔT1 after co-registration using the threshold method. **Rows ii, iv**: ΔT1 after co-registration using the saturation method. In all 5 mice, the saturation-based approach produced more accurate co-registration result than the threshold-based method.

**S-Figure 9: Consistent Detection of ESIONPs After Injection. a-e.** ΔR1 maps after injection of 1 µl of saline in 5 individual mice. **f-j.** ΔR1 maps after injection of 1 µl of ESIONPs at 0.1 mM iron concentration in 5 additional mice. **k-o.** ΔR1 maps after injection of 1 µl of ESIONPs at 0.25 mM iron concentration in 5 additional mice. The ΔR1 also reflected the R1 enhancing effect of injected ESIONPs as well as the absolute difference result shown in **Figure 7**.

**S-Figure 10: Inversion recovery T1 weighted image (IRT1WI) of naïve mice brains. column i.** IRT1WI at inversion delay time point (TI = 1300 ms). **column ii.** IRT1WI at TI = 1800ms. **column ii.** IRT1WI at TI = 1800ms. **column iii.** IRT1WI at TI = 2300ms. The gray vs white matter contrast was evident at TI = 1300 ms where it was not clear from IRT1WI at long TI, 1800 and 2300 ms.

**S-Figure 11: Inversion recovery T1 weighted images (IRT1WI) and R1 maps from MP2RAGE in control mouse brain. 1^st^ column.** IRT1WI and R1 map from 1^st^ scan. **2^nd^ column.** IRT1WI and R1 map from 2^nd^ scan which were co-registered to the 1^st^ scan. **3rd column.** absolute difference between 1^st^ and 2^nd^ scan map. **4th column.** subtraction results of 1^st^ scan from 2^nd^ scan. The R1 maps derived from MP2RAGE yielded difference and subtraction results that were highly homogeneous and close to zero over the entire mouse brain. However, the difference and subtraction results derived from IRT1WI were noisy and heterogenous for the IRT1WI approaches using both TI = 1300 ms and 3300 ms.

**S-Figure 12: Inversion recovery T1 weighted images (IRT1WI) and R1 maps from MP2RAGE in mouse brain before and after injection of 1 μl of ESIONPs at 0.1 mM [Fe]. 1^st^ column.** IRT1WI and R1 map from 1^st^ scan. **2^nd^ column.** IRT1WI and R1 map from 2^nd^ scan which were co-registered to the 1^st^ scan. The 2^nd^ scan results were obtained after intracranial injection of ESPIONs. **3rd column.** absolute difference between 1^st^ and 2^nd^ scan map. **4th column.** subtraction results of 1^st^ scan from 2^nd^ scan. The injected ESIONPs indicated by arrow were clearly detected from 2^nd^ scan R1 map (j), difference (k), and subtraction (l) map. However, none of IRT1WIs or their products showed the injected ESIONPs.

**S-Figure 13: Inversion recovery T1 weighted images (IRT1WI) and R1 maps from MP2RAGE in mouse brain before and after injection of 1 μl of ESIONPs at 0.25 mM [Fe]. 1^st^ column.** IRT1WI and R1 map from 1^st^ scan. **2^nd^ column.** IRT1WI and R1 map from 2^nd^ scan which were co-registered to the 1^st^ scan. The 2^nd^ scan results were obtained after intracranial injection of ESPIONs. **3rd column.** absolute difference between 1^st^ and 2^nd^ scan map. **4th column.** subtraction results of 1^st^ scan from 2^nd^ scan. The injected ESIONPs were detected from both IRT1WI (b) and R1 (j) map and their products (c, d, g, h, k, and l). However, the difference (c and g) and subtraction (d and h) results derived from IRT1WI suffered from additional false positive intensity enhancements in randomly scattered regions around the entire brain that could be confused with ESPIONs .

**Supplemental References**

1 Meinerz, K. *et al.* Bayesian Modeling of NMR Data: Quantifying Longitudinal Relaxation in Vivo, and in Vitro with a Tissue-Water-Relaxation Mimic (Crosslinked Bovine Serum Albumin). *Appl Magn Reson* **49**, 3-24, doi:10.1007/s00723-017-0964-z (2018).

2 Marques, J. P. *et al.* MP2RAGE, a self bias-field corrected sequence for improved segmentation and T1-mapping at high field. *Neuroimage* **49**, 1271-1281, doi:10.1016/j.neuroimage.2009.10.002 (2010).

3 Kim, B. H. *et al.* Large-scale synthesis of uniform and extremely small-sized iron oxide nanoparticles for high-resolution T1 magnetic resonance imaging contrast agents. *J Am Chem Soc* **133**, 12624-12631, doi:10.1021/ja203340u (2011).

4 Na, H. B. *et al.* Versatile PEG-derivatized phosphine oxide ligands for water-dispersible metal oxide nanocrystals. *Chem Commun (Camb)*, 5167-5169, doi:10.1039/b712721a (2007).

5 Schneider, C. A., Rasband, W. S. & Eliceiri, K. W. NIH Image to ImageJ: 25 years of image analysis. *Nat Methods* **9**, 671-675, doi:10.1038/nmeth.2089 (2012).

6 Hedayati, M. *et al.* An optimised spectrophotometric assay for convenient and accurate quantitation of intracellular iron from iron oxide nanoparticles. *Int J Hyperthermia* **34**, 373-381, doi:10.1080/02656736.2017.1354403 (2018).

S-Figure 1.


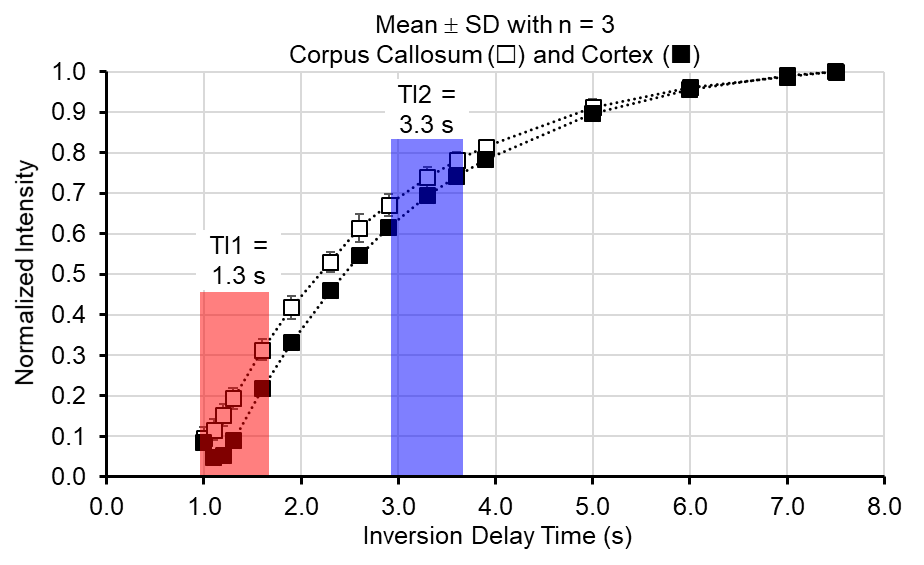


**S-Figure 1. Inversion recovery of in vivo mouse brain at 9.4T.** The inversion recovery MR data of *in vivo* mouse brain from three naïve adult mice are shown at 16 inversion delay time points, mean ± standard deviation (SD). The quantified intensity was normalized by the MR signal at 7.5 seconds inversion delay time. The red and blue boxes represent the two selected inversion delay time points for MP2RAGE. The width of each box represents segment duration time, 640 ms.

S-Figure 2.


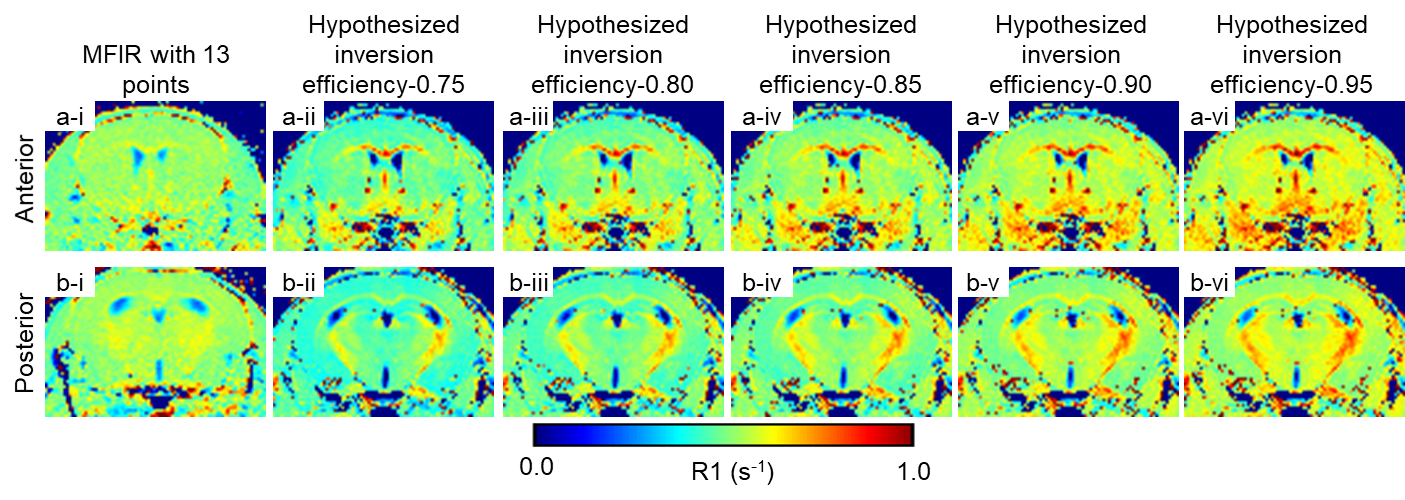


**S-Figure 2. Representative R1 maps of a naïve *in vivo* mouse brain. a-i, b-i:** R1 maps from modified fast inversion recovery at anterior (a-i) and posterior (b-i) coronal slices. **a-ii** – **a-vi:** R1 maps from MP2RAGE at an anterior coronal slice.  **b-ii** – **b-vi:** R1 maps from MP2RAGE at a posterior coronal slice. MP2RAGE data were acquired with 9 degree flip angle, 640 ms segment duration time, and TI1/TI2 (s) = 1.3/3.3. R1 maps were calculated using hypothesized inversion efficiency of 0.75 (column ii), 0.80 (column iii), 0.85 (column iv), 0.90 (column v), and 0.95 (column vi). The R1 of *in vivo* mouse brain derived from MP2RAGE increased with higher hypothesized inversion efficiency. The quantified R1 values are shown in **S-Figure 3**.

S-Figure 3.


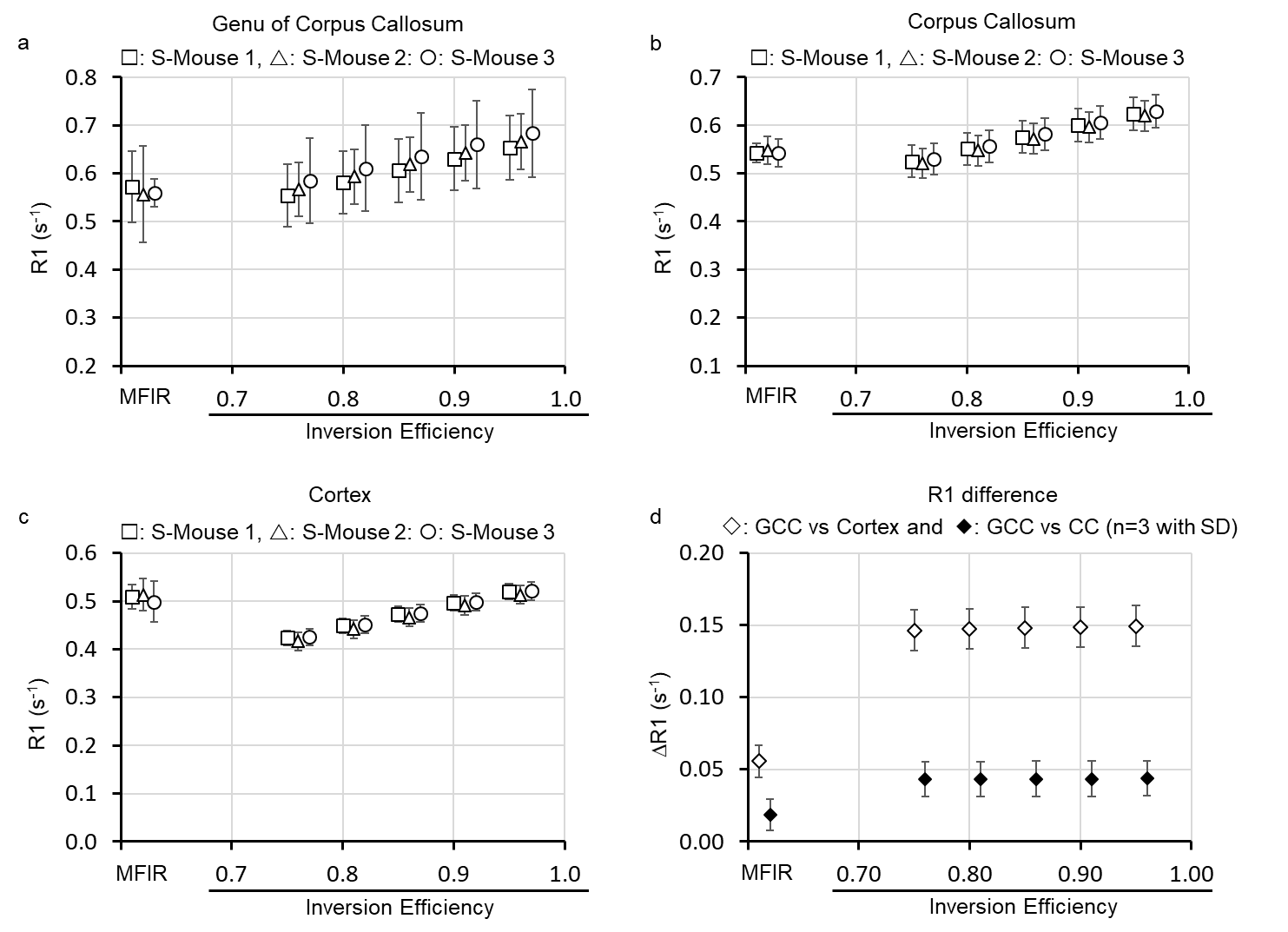


**S-Figure 3. Quantified longitudinal relaxation rate constant (R1 (s^-1^)) of three naïve *in vivo* mouse brains from modified fast inversion recovery (MFIR) and MP2RAGE.** The squares, triangles, and circles represent S-Mouse 1, S-Mouse 2, and S-Mouse 3 respectively. The error bars represent the standard deviations across voxels in each region of interest (ROI) histogram. **a.** Quantified R1 from genu of corpus callosum (GCC). **b.** Quantified R1 from mid-corpus callosum (CC). **c.** Quantified R1 from cortex. The R1 of *in vivo* mouse brains derived from MP2RAGE increased with higher hypothesized inversion efficiency in all brain regions. **d.** R1 differences between tissues: GCC vs. cortex (white diamonds) and GCC vs. CC (black diamonds). Compared to the R1 from MFIR, the MP2RAGE overestimated the R1 differences between different brain tissues. The overestimation of R1 differences was largely preserved for all hypothesized inversion efficiencies. Based on the results, an inversion efficiency of 0.85 for MP2RAGE was used for all R1 calculations in the entire study.

S-Figure 4.


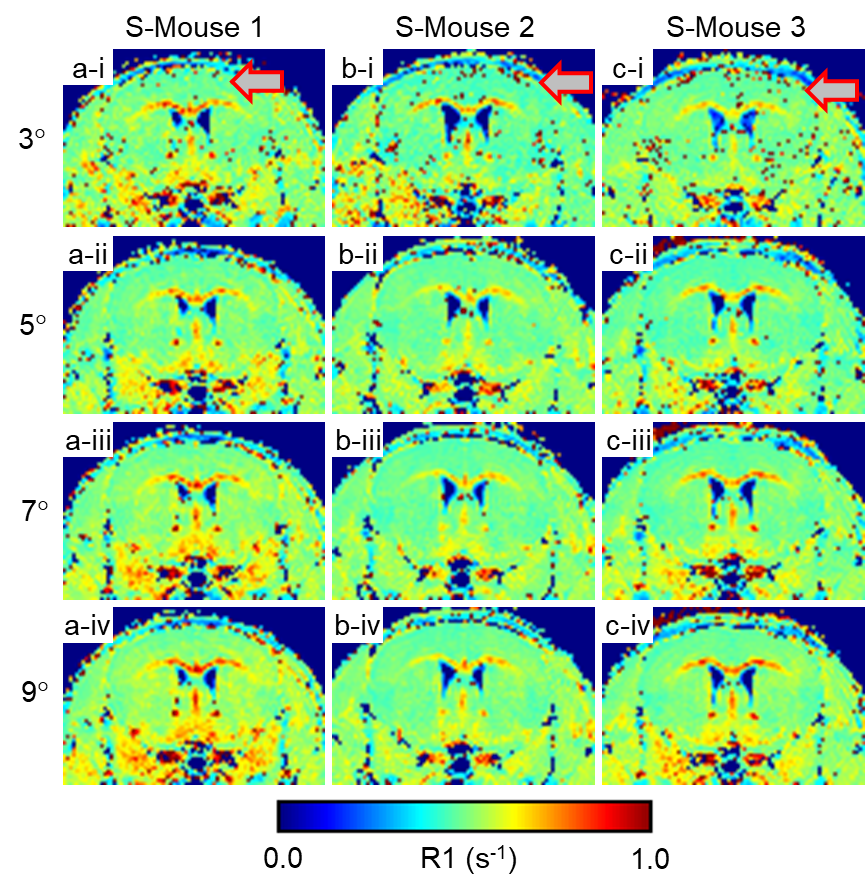


**S-Figure 4. Optimization of flip angle for MP2RAGE.** The R1 maps were calculated using 0.85 inversion efficiency. Acquisition parameters included 640 ms segment duration and TI1/TI2 (s) = 1.3/3.3. **row i:** R1 maps of three naïve *in vivo* mouse brains with flip angle 3 degrees. **row ii:** R1 maps of three naïve *in vivo* mouse brains with flip angle 5 degrees. **row iii:** R1 maps of three naïve *in vivo* mouse brains with flip angle 7 degrees. **row iv:** R1 maps of three naïve *in vivo* mouse brains with flip angle 9 degrees. Overall, the R1 values were well preserved from 3 to 9 degrees for all three mice. The R1 maps with 3 degree flip angle had random artifacts, indicated by arrows, due to low SNR. No evidence or R1 deviation was observed at 9 degree flip angle compared to R1 at lower flip angles.

S-Figure 5.


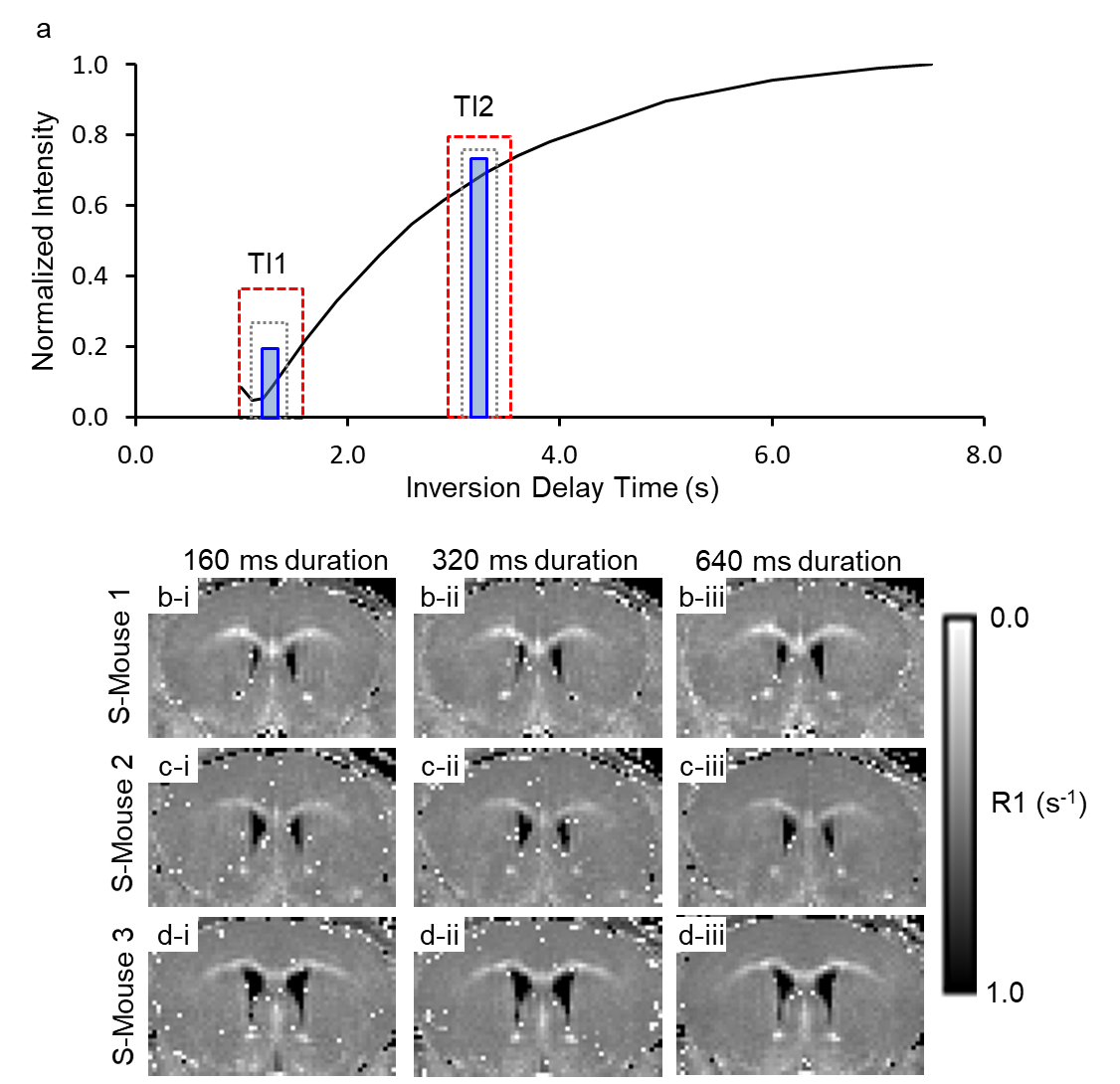


**S-Figure 5. Optimization of segment duration for MP2RAGE.** The R1 maps were derived from MP2RAGE data using 0.85 inversion efficiency, 9 degree flip angle, and TI1/TI2 (s) = 1.3/3.3. **a.** 160 (blue box with solid line), 320 (gray box dotted line), and 640 ms (red box with dashed line) segment duration for inversion recovery acquisition of MP2RAGE data for *in vivo* mouse brain. **column i.** R1 maps from 160 ms segment duration. **column ii.** R1 maps from 320 ms segment duration. **column iii.** R1 maps from 640 ms segment duration. The R1 values were largely preserved for all three segment duration times, but noise was lower in with longer segment duration.

S-Figure 6.


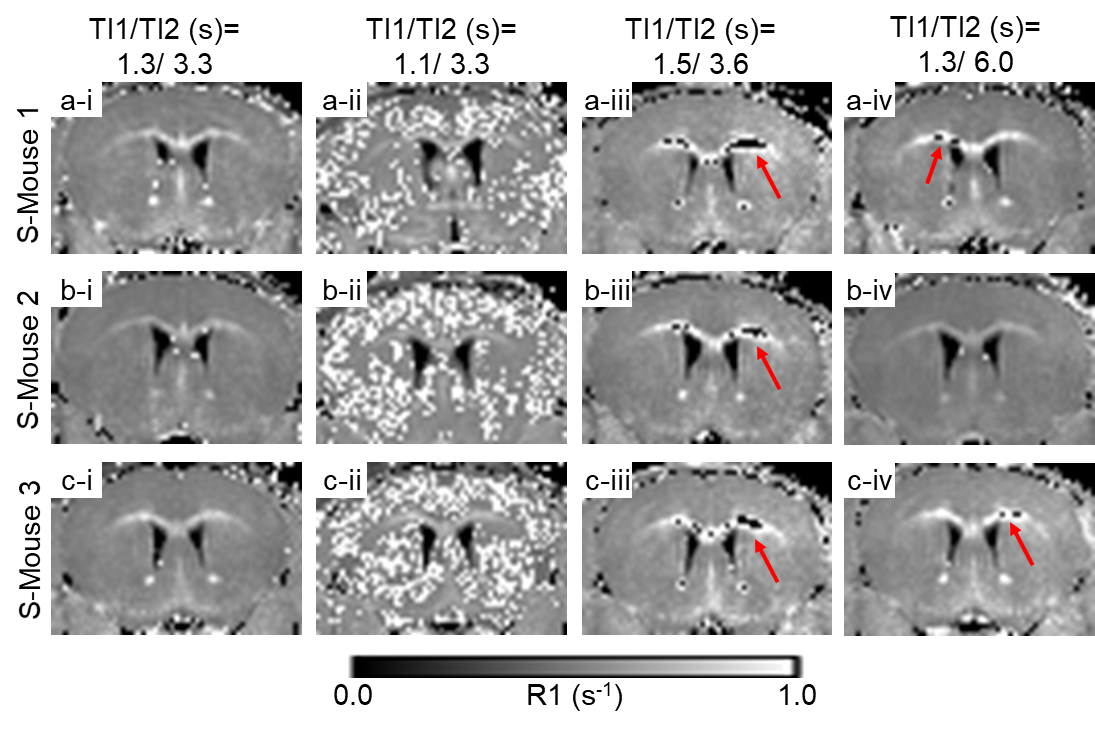


**S-Figure 6. Optimization of TI1 and TI2 for MP2RAGE.** The R1 maps were derived from MP2RAGE data using 0.85 inversion efficiency, 9 degree flip angle, and 640 ms segment duration time. **column i.** R1 maps from TI1/TI2 (s) = 1.3/3.3. The R1 maps with TI1/TI2 (s) = 1.3/3.3 show good quality of *in vivo* mouse longitudinal relaxation rate constant measures. **column ii.** R1 maps from TI1/TI2 (s) = 1.1/3.3. The R1 maps with TI1/TI2 (s) = 1.1/3.3 suffer from overestimated R1 artifacts, apparent as bright spots. **column iii.** R1 maps from TI1/TI2 (s) = 1.5/3.6. The R1 maps with TI1/TI2 (s) = 1.5/3.6 suffer from underestimated R1 artifact indicated by arrows. **column iv.** R1 maps from TI1/TI2 (s) = 1.3/6.0. Similar artifacts are seen from the R1 maps with TI1/TI2 (s) = 1.3/6.0.

S-Figure 7.


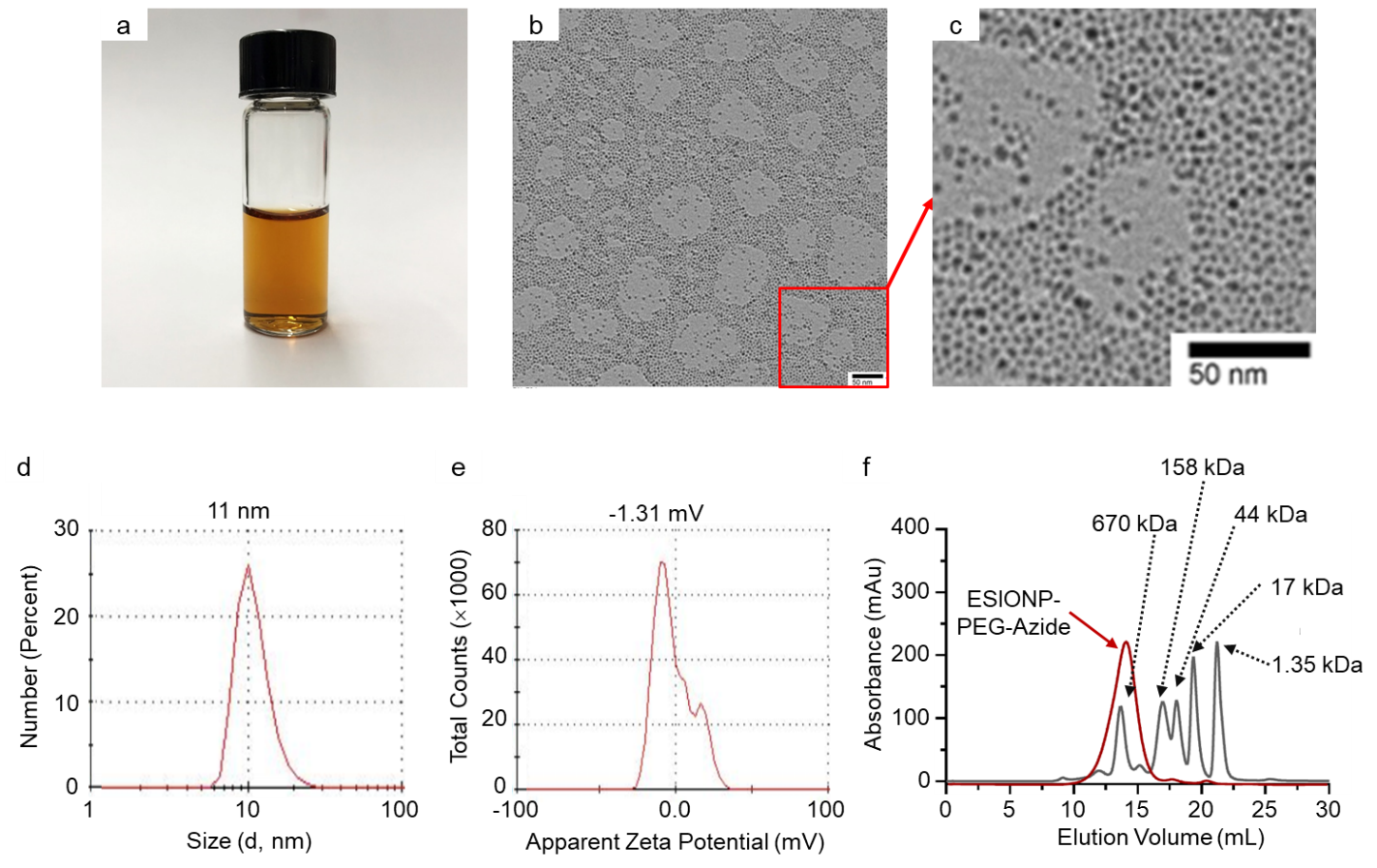


**S-Figure 7. Characterization of Extremely Small Iron Oxide Nanoparticles (ESIONPs).** **a.** Photograph of the ESIONPs with polyethylene glycol coating and an azide functional group (ESIONP-PEG-Azide) suspended in 0.9% NaCl, 0.05% v/v Tween 80 solution. The ESIONPs were highly soluble in aqueous solutions. **b.** TEM Image of ESIONP cores. **c.** Close up view of the box in panel b indicating homogenous iron oxide core sizes. **d.** Dynamic light scattering (DLS) size distribution of ESIONP-PEG-Azide, mean value of 11.0 nm. **e.** Zeta potential of ESIONP-PEG-Azide, mean value of -1.31 mV. **f.** Size exclusion chromatography of ESIONP-PEG-Azide (red curve, red arrow) in human plasma with size standard references (gray curve, dotted arrows). The ESIONPs had homogenous hydrodynamic size and no significant fouling.

S-Figure 8.


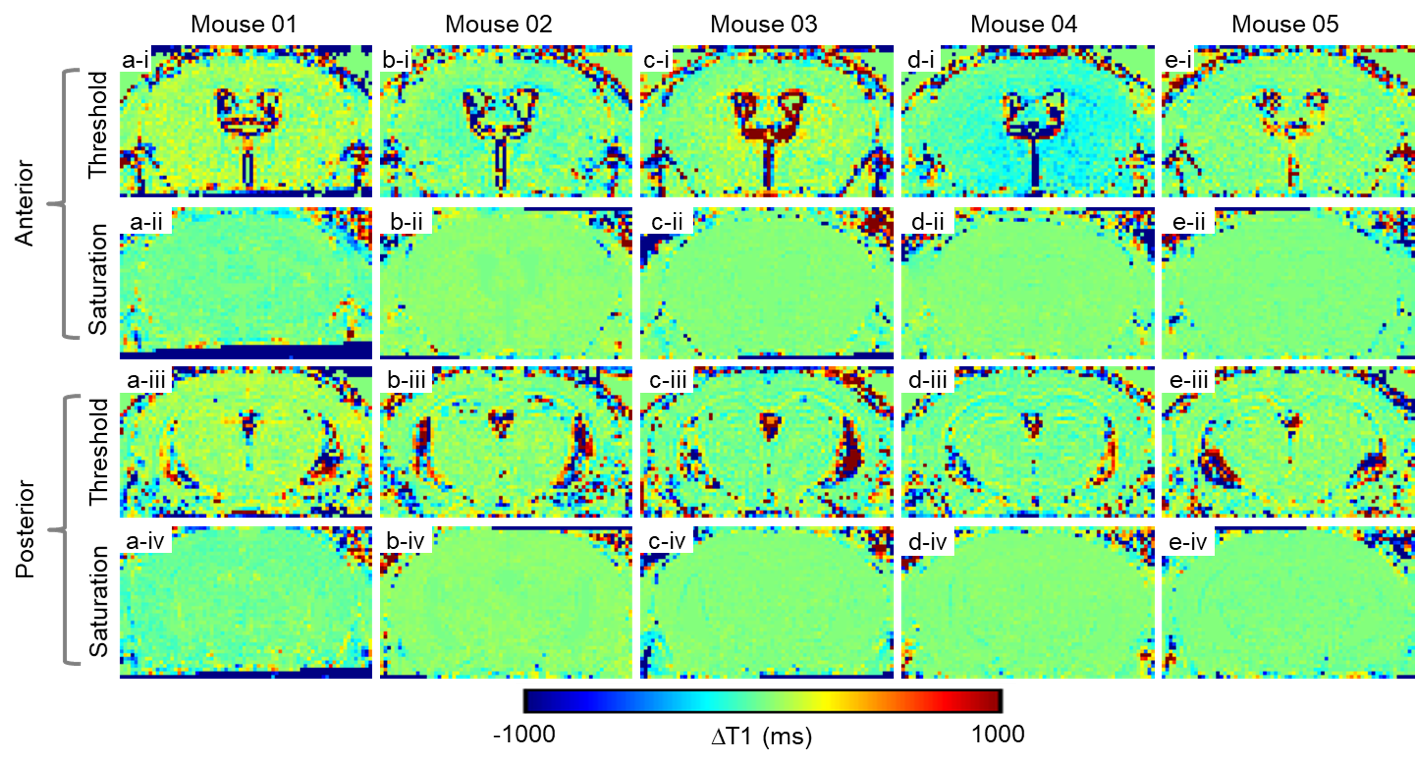


**S-Figure 8: ΔT1 of first scan from second scan in 5 control mice**. **a-e:** Each column represents an individual mouse. **Rows i, iii**: absolute ΔT1 after co-registration using the threshold method. **Rows ii, iv**: ΔT1 after co-registration using the saturation method. In all 5 mice, the saturation-based approach produced more accurate co-registration result than the threshold-based method.

S-Figure 9.


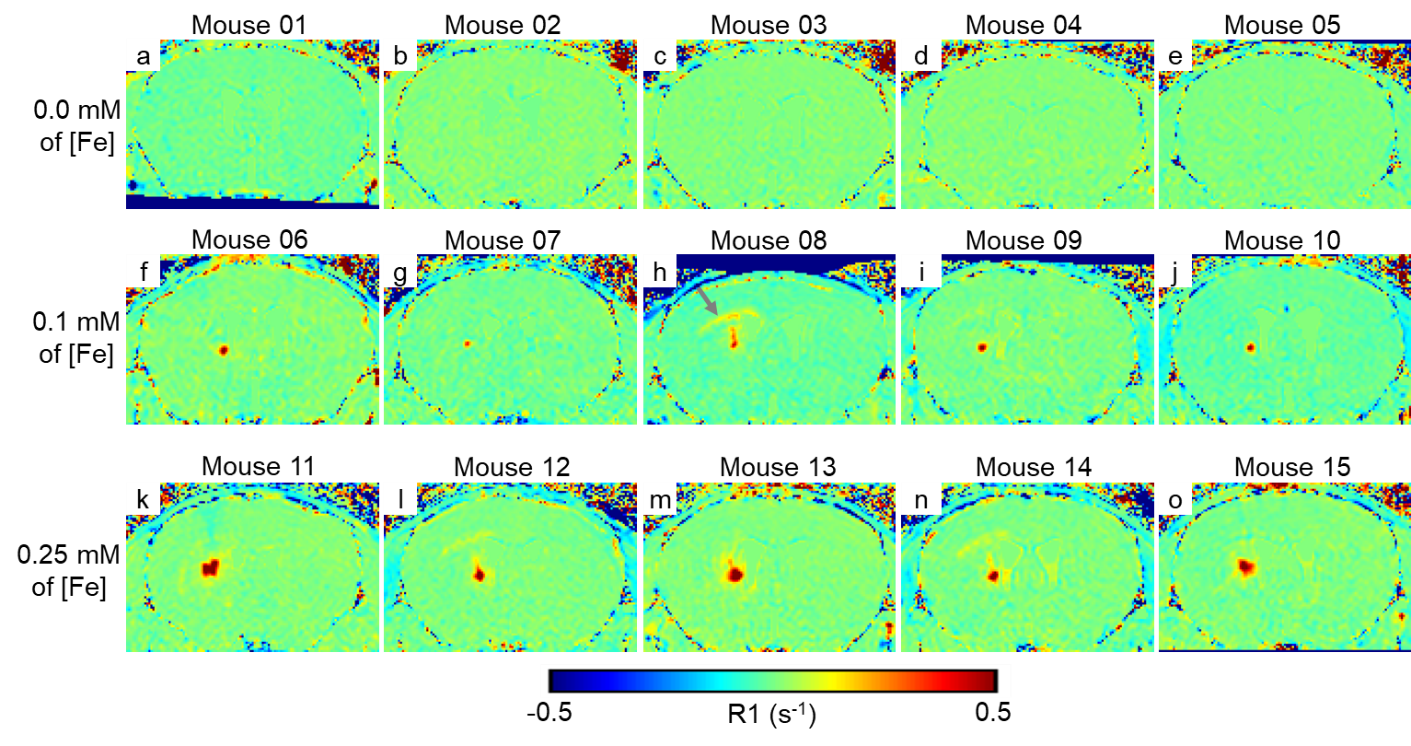


**S-Figure 9: Consistent Detection of ESIONPs After Injection. a-e.** ΔR1 maps after injection of 1 µl of saline in 5 individual mice. **f-j.** ΔR1 maps after injection of 1 µl of ESIONPs at 0.1 mM iron concentration in 5 additional mice. **k-o.** ΔR1 maps after injection of 1 µl of ESIONPs at 0.25 mM iron concentration in 5 additional mice. The ΔR1 also reflected the R1 enhancing effect of injected ESIONPs as well as the absolute difference result shown in **Figure 7**.

S-Figure 10.


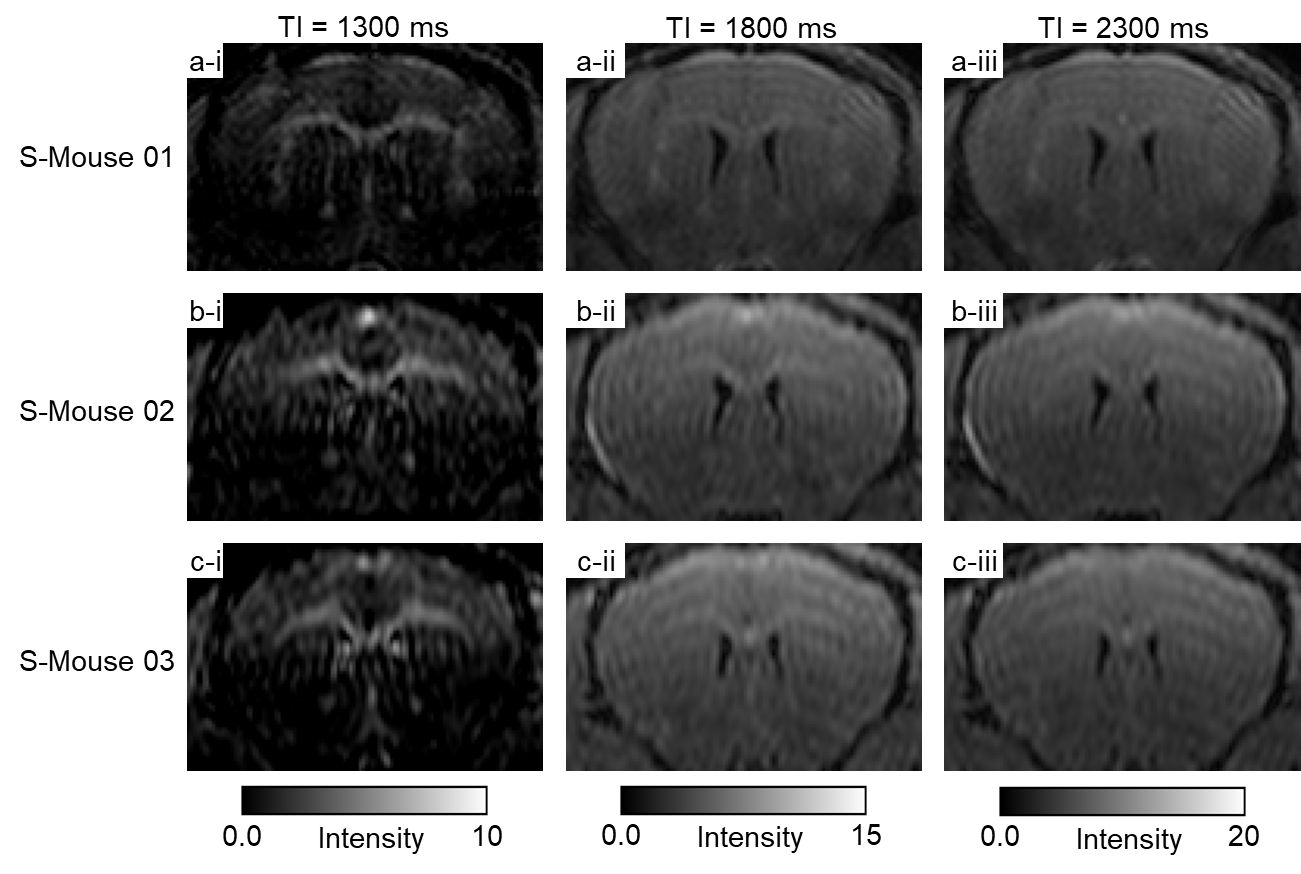


**S-Figure 10: Inversion recovery T1 weighted image (IRT1WI) of naïve mice brains. column i.** IRT1WI at inversion delay time point (TI = 1300 ms). **column ii.** IRT1WI at TI = 1800ms. **column ii.** IRT1WI at TI = 1800ms. **column iii.** IRT1WI at TI = 2300ms. The gray vs white matter contrast was evident at TI = 1300 ms where it was not clear from IRT1WI at long TI, 1800 and 2300 ms.

S-Figure 11.


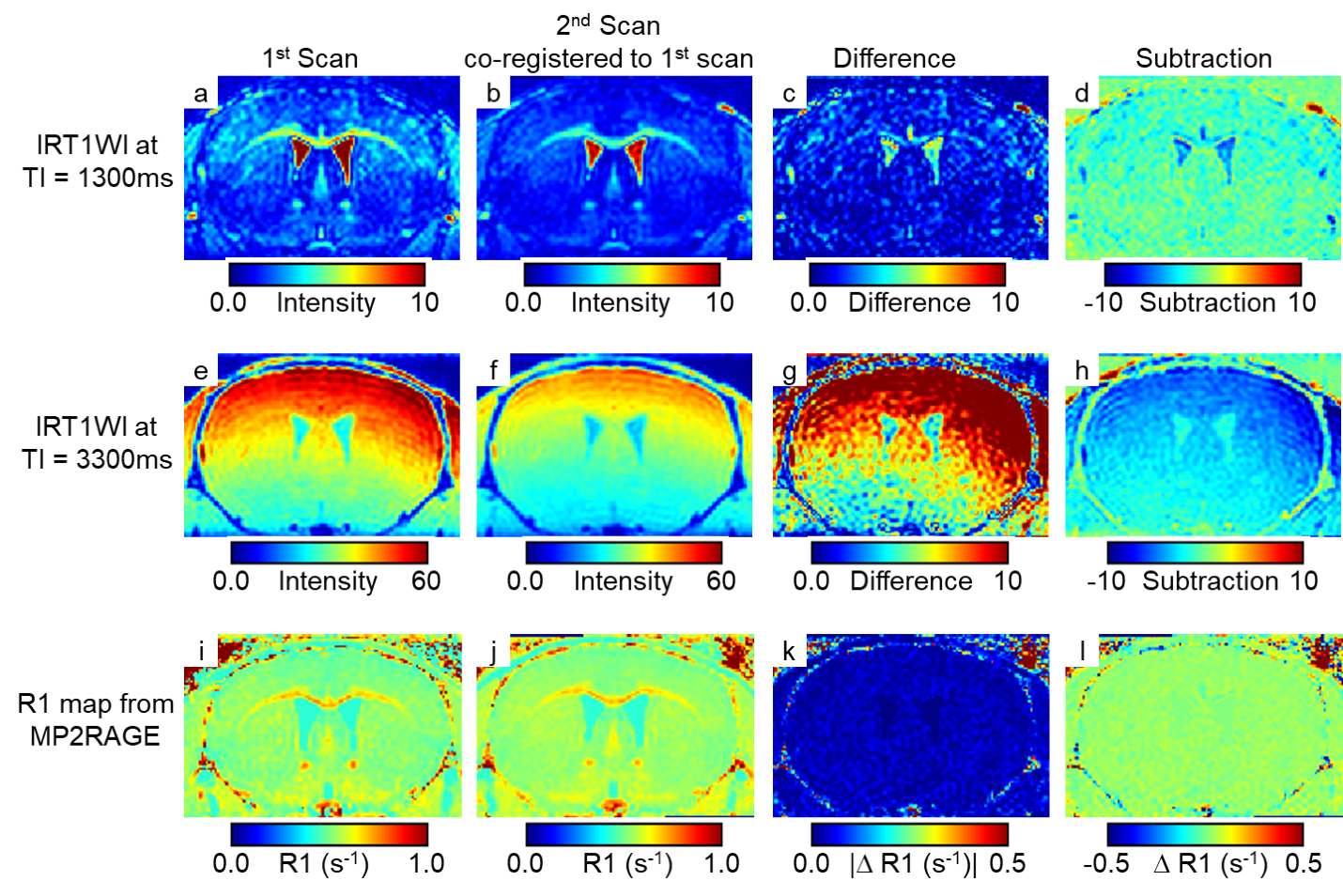


**S-Figure 11: Inversion recovery T1 weighted images (IRT1WI) and R1 maps from MP2RAGE in control mouse brain. 1^st^ column.** IRT1WI and R1 map from 1^st^ scan. **2^nd^ column.** IRT1WI and R1 map from 2^nd^ scan which were co-registered to the 1^st^ scan. **3rd column.** absolute difference between 1^st^ and 2^nd^ scan map. **4th column.** subtraction results of 1^st^ scan from 2^nd^ scan. The R1 maps derived from MP2RAGE yielded difference and subtraction results that were highly homogeneous and close to zero over the entire mouse brain. However, the difference and subtraction results derived from IRT1WI were noisy and heterogenous for the IRT1WI approaches using both TI = 1300 ms and 3300 ms.

S-Figure 12.


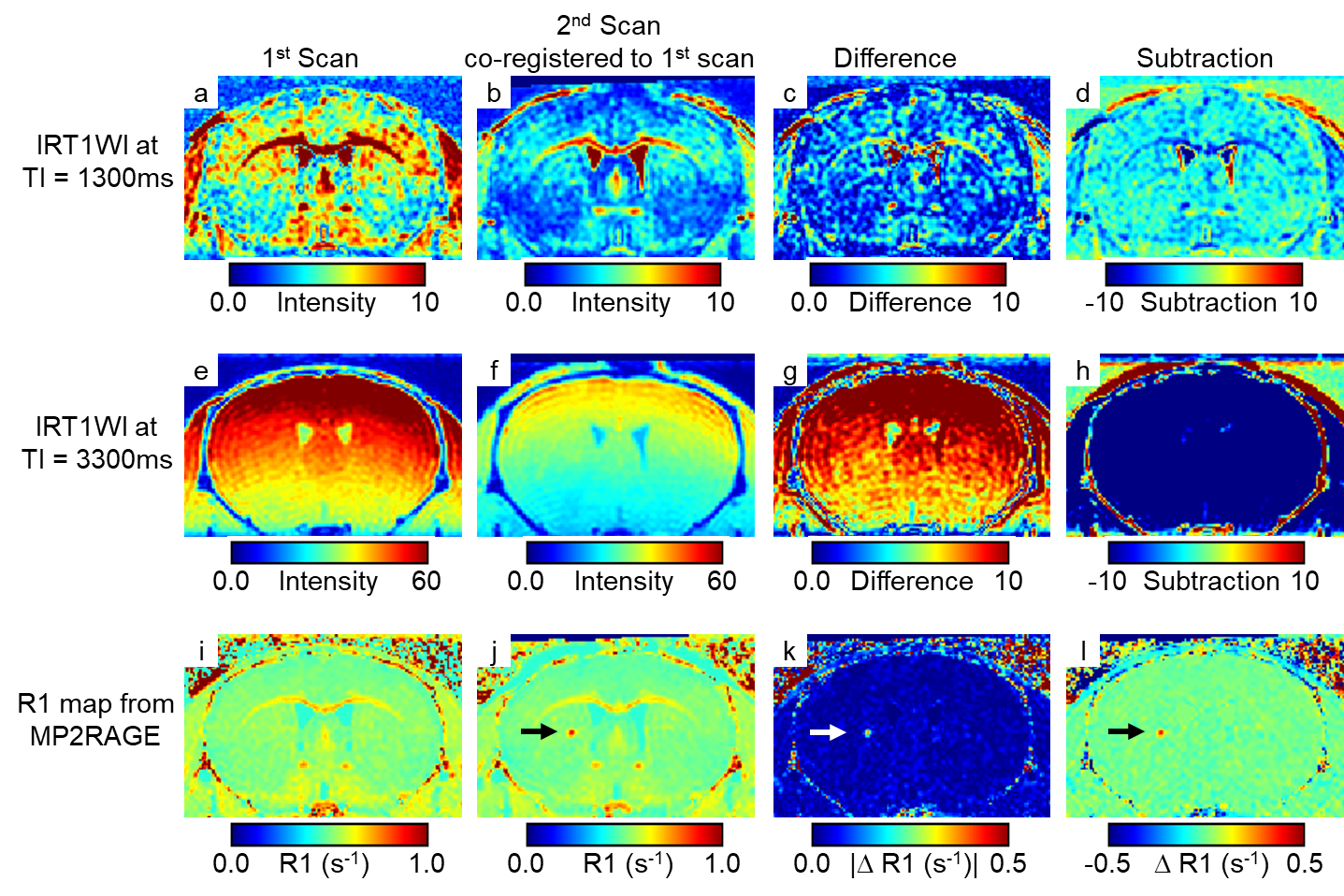


**S-Figure 12: Inversion recovery T1 weighted images (IRT1WI) and R1 maps from MP2RAGE in mouse brain before and after injection of 1 μl of ESIONPs at 0.1 mM [Fe]. 1^st^ column.** IRT1WI and R1 map from 1^st^ scan. **2^nd^ column.** IRT1WI and R1 map from 2^nd^ scan which were co-registered to the 1^st^ scan. The 2^nd^ scan results were obtained after intracranial injection of ESPIONs. **3rd column.** absolute difference between 1^st^ and 2^nd^ scan map. **4th column.** subtraction results of 1^st^ scan from 2^nd^ scan. The injected ESIONPs indicated by arrow were clearly detected from 2^nd^ scan R1 map (j), difference (k), and subtraction (l) map. However, none of IRT1WIs or their products showed the injected ESIONPs.

S-Figure 13.


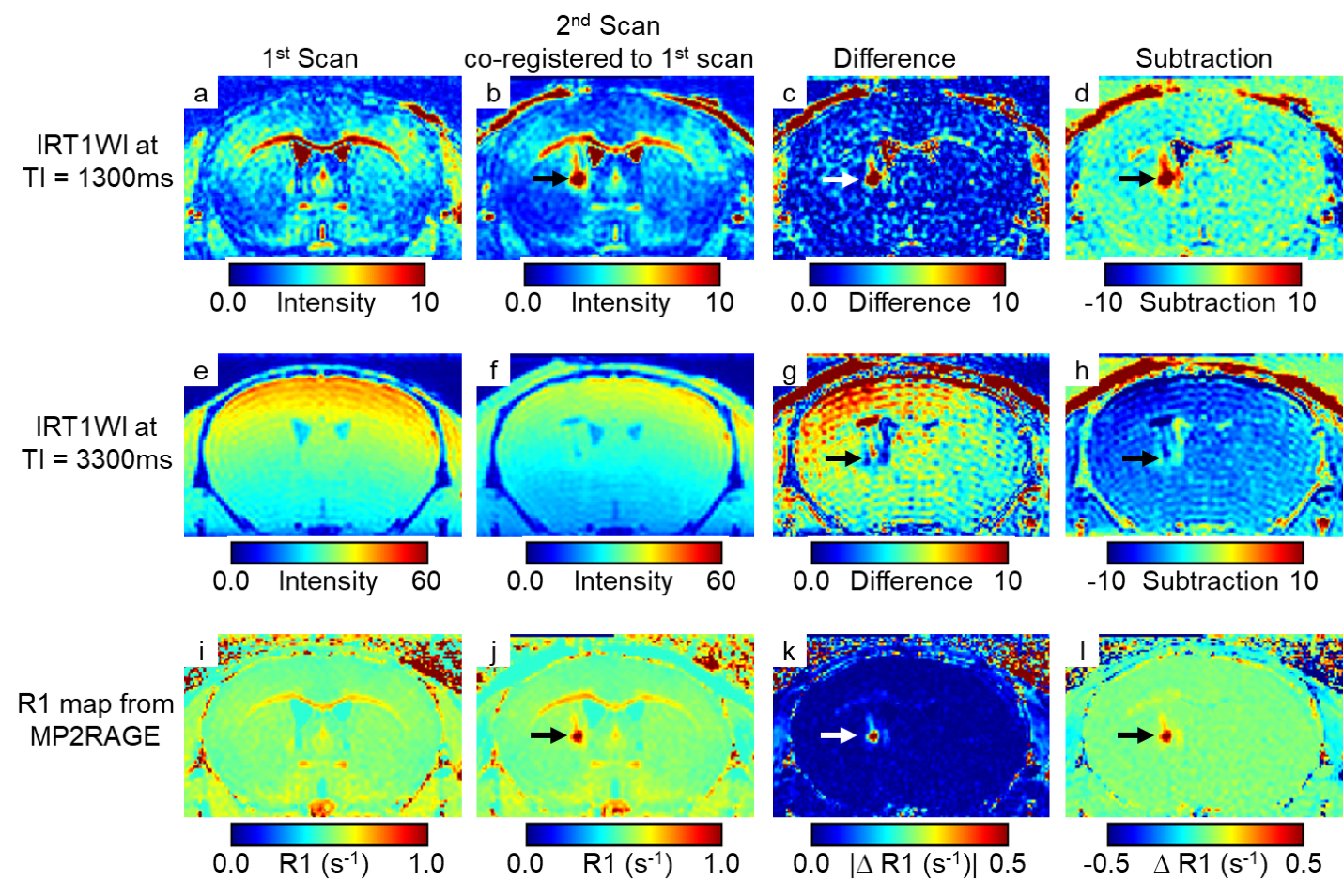


**S-Figure 13: Inversion recovery T1 weighted images (IRT1WI) and R1 maps from MP2RAGE in mouse brain before and after injection of 1 μl of ESIONPs at 0.25 mM [Fe]. 1^st^ column.** IRT1WI and R1 map from 1^st^ scan. **2^nd^ column.** IRT1WI and R1 map from 2^nd^ scan which were co-registered to the 1^st^ scan. The 2^nd^ scan results were obtained after intracranial injection of ESPIONs. **3rd column.** absolute difference between 1^st^ and 2^nd^ scan map. **4th column.** subtraction results of 1^st^ scan from 2^nd^ scan. The injected ESIONPs were detected from both IRT1WI (b) and R1 (j) map and their products (c, d, g, h, k, and l). However, the difference (c and g) and subtraction (d and h) results derived from IRT1WI suffered from additional false positive intensity enhancements in randomly scattered regions around the entire brain that could be confused with ESPIONs .
